# Red light induces salicylic acid accumulation by activating CaHY5 to enhance pepper resistance against *Phytophthora capsici*

**DOI:** 10.1093/hr/uhad213

**Published:** 2023-10-17

**Authors:** Youxin Yang, Yu Li, Yelan Guang, Jinhui Lin, Yong Zhou, Ting Yu, Fei Ding, Yanfeng Wang, Jinyin Chen, Yanhong Zhou, Fengfeng Dang

**Affiliations:** Jiangxi Key Laboratory for Postharvest Technology and Nondestructive Testing of Fruits & Vegetables, Collaborative Innovation Center of Post-Harvest Key Technology and Quality Safety of Fruits and Vegetables, College of Agronomy, Jiangxi Agricultural University, Nanchang, 330045, China; Jiangxi Key Laboratory for Postharvest Technology and Nondestructive Testing of Fruits & Vegetables, Collaborative Innovation Center of Post-Harvest Key Technology and Quality Safety of Fruits and Vegetables, College of Agronomy, Jiangxi Agricultural University, Nanchang, 330045, China; Jiangxi Key Laboratory for Postharvest Technology and Nondestructive Testing of Fruits & Vegetables, Collaborative Innovation Center of Post-Harvest Key Technology and Quality Safety of Fruits and Vegetables, College of Agronomy, Jiangxi Agricultural University, Nanchang, 330045, China; Fruit Research Institute, Fujian Academy of Agricultural science, Fuzhou 350013, China; Jiangxi Key Laboratory for Postharvest Technology and Nondestructive Testing of Fruits & Vegetables, Collaborative Innovation Center of Post-Harvest Key Technology and Quality Safety of Fruits and Vegetables, College of Agronomy, Jiangxi Agricultural University, Nanchang, 330045, China; Jiangxi Key Laboratory for Postharvest Technology and Nondestructive Testing of Fruits & Vegetables, Collaborative Innovation Center of Post-Harvest Key Technology and Quality Safety of Fruits and Vegetables, College of Agronomy, Jiangxi Agricultural University, Nanchang, 330045, China; School of Life Sciences, Liaocheng University, Liaocheng 252000, China; Shaanxi Key Laboratory of Chinese Jujube, Yan’an University, Yan’an, Shaanxi 716000, China; Jiangxi Key Laboratory for Postharvest Technology and Nondestructive Testing of Fruits & Vegetables, Collaborative Innovation Center of Post-Harvest Key Technology and Quality Safety of Fruits and Vegetables, College of Agronomy, Jiangxi Agricultural University, Nanchang, 330045, China; Department of Horticulture, Zijingang Campus, Zhejiang University, Yuhangtang Road 866, Hangzhou, 310058, China; Shaanxi Key Laboratory of Chinese Jujube, Yan’an University, Yan’an, Shaanxi 716000, China

## Abstract

Pepper (*Capsicum annuum* L.) is frequently challenged by various pathogens, among which *Phytophthora capsici* is the most devastating to pepper production. Red light signal acts as a positive induction of plant resistance against multiple pathogens. However, little is known about how the red light signal affects pepper resistance to *P. capsici* infection (PCI). Here, we report that red light regulates salicylic acid (SA) accumulation by activating elongated hypocotyl5 (CaHY5), a basic leucine zipper (bZIP) transcription factor, thereby decreasing pepper susceptibility to PCI. Exogenous SA treatment reduced pepper susceptibility to PCI, while silencing of *CaPHYB* (a red light photoreceptor) increased its susceptibility. PCI significantly induced *CaHY5* expression, and silencing of *CaHY5* reduced SA accumulation, accompanied by decreases in the expression levels of *phenylalanine ammonia-lyase 3 (CaPAL3)*, *CaPAL7*, **pathogenesis-related 1* (*CaPR1*)*, and *CaPR1L*, which finally resulted in higher susceptibility of pepper to PCI. Moreover, CaHY5 was found to activate the expression of *CaPAL3* and *CaPAL7*, which are essential for SA biosynthesis, by directly binding to their promoters. Further analysis revealed that exogenous SA treatment could restore the resistance of *CaHY5*­silenced pepper plants to PCI. Collectively, this study reveals a critical mechanism through which red light induces SA accumulation by regulating CaHY5­mediated *CaPAL3* and *CaPAL7* expression, leading to enhanced resistance to PCI. Moreover, red light-induced CaHY5 regulates pepper resistance to PCI, which may have implications for PCI control in protected vegetable production.

## Introduction

Plants, unlike mobile animals, are sessile organisms and have evolved various strategies to adapt to the ever-changing environment. During early pathogen infections, light acts as an important environmental signal to increase plant resistance, in addition to providing energy for photosynthesis [1–3]. Accumulating evidence has demonstrated that red light is the most effective type of light to enhance plant resistance to pathogens [4, 5]. For example, compared with white light, red light can enhance tomato resistance to the leaf disease [6], and improve tomato systemic resistance to root-knot nematode in roots [7]. Red light can also inhibit damping­off disease in pepper seedlings [8]. Moreover, previous studies have identified the red (R) light and far­red (FR) photoreceptors, including five members from phyA to phyE. Among them, *Arabidopsis* light photoreceptor phyB plays a critical role in the defense against pathogens by interacting with salicylic acid (SA) and jasmonic acid (JA)­mediated immunities [9]. Compared with wild-type plants, rice phytochrome triple mutants (*phyAphyBphyC*) were more susceptible to blast disease [10]. In tobacco, phyA and phyB play positive roles in regulating defense against invading viruses (*Chilli veinal mottle virus*) [11]. Although red light has been found to enhance plant resistance to pathogen attack, it remains unclear whether and how red light is involved in regulating pepper resistance to *Phytophthora capsici*, an oomycete pathogen.

Elongated hypocotyl5 (HY5) is a basic leucine zipper (bZIP) type transcription factor, that accumulates through multiple photoreceptors (phyA and phyB), and functions in regulating photomorphogenesis, pigment biosynthesis, and defense responses [12, 13]. In the promoters of light- and hormone-responsive genes, HY5 preferentially binds to either the G­box (CACGTG) and/or the E­box (CAATTG) motif [13]. Additionally, red light induces the expression of *HY5*, which directly activates the *Enhanced Disease Susceptibility* 1 (*EDS1*) gene and enhances pathogens defense [14, 15]. Recently, HY5 was found to directly modulate the expression of defense-related genes in host plants in response to *Hyaloperonospora arabidopsidis* (*Hpa*) infection. Moreover, by decreasing the deoxyribonucleic acid (DNA) binding activity of HY5, a conserved RxLR effector, HaRxLL470, inhibits defense-related genes transcription [16]. Although HY5-regulated plant immunity has been reported, it remains to be determined whether and how CaHY5 activates pepper immunity to *Phytophthora capsici* infection (PCI).

In nature, one major challenge for horticultural crops is the attack by various microbial pathogens in the agricultural production process, particularly soil-borne pathogens [17]. In crop breeding, disease resistance of crops has been greatly improved through long-term selection and improvement. However, crops are still faced with serious threats from diseases due to the co­evolution between pathogens and plants [18, 19]. Traditionally, plants have formed sophisticated immune systems, allowing them to resist the infection of pathogens or slow down the disease progression. In plants, for a proper response to pathogen attack, pattern recognition receptors (PRRs) on the surface of cells sense microbe-associated molecular patterns (MAMPs), triggering pattern-triggered immunity (PTI), which is in fact a PRR-mediated innate immunity.

However, a number of effectors are secreted by pathogens into host cells to suppress the PTI. Nucleotide-binding leucine-rich repeat (NB-LRR; NLRs) proteins recognize certain effectors, resulting in effector-triggered immunity (ETI), which can be regarded as an NLR-mediated innate immunity [18]. Although ETI has a strong and fast response to pathogen infection, both ETI and PTI use the same signaling components, such as phytohormones SA, JA, and ET [20]. Among them, SA signal-induced immunity is required for resistance against biotrophic pathogens [21]. SA-mediated plant immunity functions through the transcriptional activator NPR1 (Non-expression of Pathogenesis Related 1), and transcriptional repressors NPR3 and NPR4. These proteins bind SA and are also SA receptors [22, 23]. In addition, the expression of SA-responsive genes is modulated by various transcription factors (TFs).

A major horticultural crop around the world, pepper (*Capsicum annuum* L.), is often cultivated and produced in warm seasons and greenhouses with a suitable growing temperature range of 20–30°C. Pepper is frequently attacked by various pathogens, particularly the soil-borne pathogen *P. capsici*, which causes Phytophthora blight disease with the most serious threat to pepper growth and production. Moreover, greenhouse cultivation is the most common and effective method of pepper production worldwide. Nevertheless, low light quality and high humidity tend to decrease the resistance of pepper to *P. capsici* infection (PCI) under greenhouse conditions, which seriously affects the production of pepper. Therefore, a better understanding of the molecular and genetic basis for pepper resistance to PCI is required for the development of effective countermeasures including molecular breeding and light quality manipulation.

In this study, we report on the critical role for CaHY5 TF in red light-induced SA accumulation and expression of SA­responsive genes such as *CaPR1* and *CaPR1L* during PCI in pepper. Red light induced the expression of *CaHY5*, which further directly activated the expression of *CaPAL3* and *CaPAL7*, two *PAL* genes playing critical roles in the biosynthesis and accumulation of SA, resulting in the accumulation of SA and up­regulation of the expression of SA-responsive *CaPR1* and *CaPR1L* genes, and thereby increasing pepper resistance to PCI. These findings shed new light on the mechanism involved in pepper resistance to PCI and highlight the key role of red light and CaHY5 in regulating SA accumulation in pepper immune responses.

## Results

### Red light induces SA biosynthesis and *Phytophthora capsici* resistance in pepper

**Figure 1:**
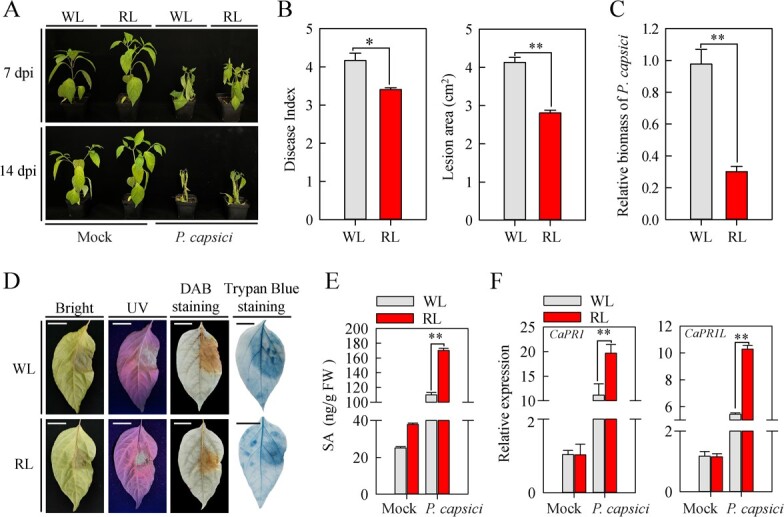
Red light decreases pepper susceptibility to *Phytophthora capsici* infection (PCI). **A** Resistance level analysis of pepper plants to PCI under the white light (WL) and red light (RL) treatments. Photographs were acquired at 7 and 14 days post infection (dpi). **B** Red light decreases the disease index (7 dpi) and lesion area (4 dpi) of *P. capsici*-infected pepper plants. **C***P. capsici* biomass in infected pepper leaves under the white light and red light treatments at 4 dpi determined by qRT-PCR. **D** Red light decreases H_2_O_2_ levels and cell death of *P. capsici*-infected leaves in pepper. Images were acquired at 4 dpi. Scale bars represent 1 cm. **E** Contents of endogenous SA in pepper leaves were increased by exposure of plants to red light at 4 dpi with *P. capsici*. **F** Expression levels of SA-responsive genes such as *CaPR1* and *CaPR1L* were up-regulated by exposure of pepper plants to red light at 4 dpi. Plants were infected with Mock and *P. capsici*, ddH_2_O was represented as Mock. Data represent the mean ± SE (*n* = 3).

To determine whether red light can induce resistance to *P. capsici* infection (PCI) in pepper, we investigated the effect of red light on the resistance of the six-leaf stage pepper plants to PCI after 7 and 14 days of exposure to either red light or white light (WL, 200 μmol m^−2^ s^−1^). All pepper plants exhibited some disease symptoms after PCI. However, red light (RL) decreased the susceptibility compared with white light at 7 and 14 days post infection (dpi), as indicated by a lower disease index and smaller lesion area (Fig. 1A and B). At 4 dpi, we compared the biomass of *P. capsici* on the leaves of white light- and red light-treated pepper plants. The biomass of *P. capsici* on red light-treated leaves significantly decreased relative to that on white light-treated leaves, indicating that the disease severity of white light-treated plants was much greater than that of red light-treated plants (Fig. 1C).

In addition, PCI induced disease-caused H_2_O_2_ accumulation (monitored through 3,3′­diaminobenzidine (DAB) staining) and cell death (monitored through trypan blue staining) in both white light and red light treated pepper plants, but red light treatment resulted in relatively lower H2O2 accumulation and less extensive cell death (Fig. 1D) than white light at 4 dpi. Based on these results, red light enhanced pepper resistance to PCI.

To reveal the molecular mechanism through which red light regulates pepper resistance to PCI, we determined the free SA content. As a result, red light increased SA accumulation at 4 dpi in pepper (Fig. 1E). To confirm this result, the expression of *CaPR1* and *CaPR1L*, two SA-responsive genes, were measured in pepper plants at 4 dpi under treatment of white light and red light through RT-qPCR assay. The results showed that PCI induced much higher expression of *CaPR1* and *CaPR1L* in red light-treated pepper plants than in white light-treated pepper plants (Fig. 1F). Consistently, the susceptibility of pepper plants to PCI was decreased by exogenous application of SA, as indicated by fewer disease symptoms and lower disease index (Fig. S1A, see online supplementary material). Moreover, PCI led to a significantly smaller lesion area on SA-treated pepper leaves than the control (Fig. S1B, see online supplementary material), as well as lower biomass of *P. capsici* (Fig. S1C, see online supplementary material). Similarly, PCI induced less H_2_O_2_ accumulation and cell death on the SA-treated pepper leaves than the control (Fig. S1D, see online supplementary material). Overall, these results demonstrated that red light enhances resistance to PCI in pepper by accumulating SA and promoting SA­responsive gene expression.

### Red light photoreceptor CaPHYB2 positively regulates pepper resistance to PCI

We next attempted to identify the genes acting downstream of red light to modulate SA­mediated PCI resistance of pepper. The red light photoreceptor CaPHYB2 was the first candidate gene based on observation. First, genome-wide identification revealed that five putative photoreceptor proteins were identified in the pepper (*C. annuum*) genome, and named *CaPHYA*, *CaPHYB1*, *CaPHYB2*, *CaPHYE*, and *CaPHYF* (Fig. S2A and B, Table S1 see online supplementary material) based on their sequence homology and phylogenetic analysis with *Arabidopsis*, tomato, and rice photoreceptor proteins (Fig. S2C, see online supplementary material). Second, pepper CaPHYB2 showed high sequence similarity to AtPHYB (Fig. S3, see online supplementary material), a red light photoreceptor known to play a predominant role in perceiving red light signals in *Arabidopsis*. Third, to verify the role of *CaPHYB2* in pepper during PCI, we generated *CaPHYB2*-silenced pepper plants via VIGS with the *TRV:phyb2* construct, in which the expression of *CaPHYB2* was reduced (Fig. S4A, see online supplementary material). The *CaPHYB2*-silenced pepper plants were more susceptible to PCI at 9 dpi than an infection with the *TRV:00* empty vector in control plants (Fig. S4B, see online supplementary material), as indicated by significantly higher disease index (Fig. S4C, see online supplementary material), larger lesion area (Fig. S4D, see online supplementary material), and higher biomass of *P. capsici* (Fig. S4E, see online supplementary material), as well as more H_2_O_2_ accumulation and cell death (Fig. S4F, see online supplementary material). Together, these results demonstrated that the red light photoreceptor *CaPHYB2* positively modulates pepper resistance to PCI.

### *CaHY5* is induced by red light and positively regulates pepper resistance to PCI

**Figure 2:**
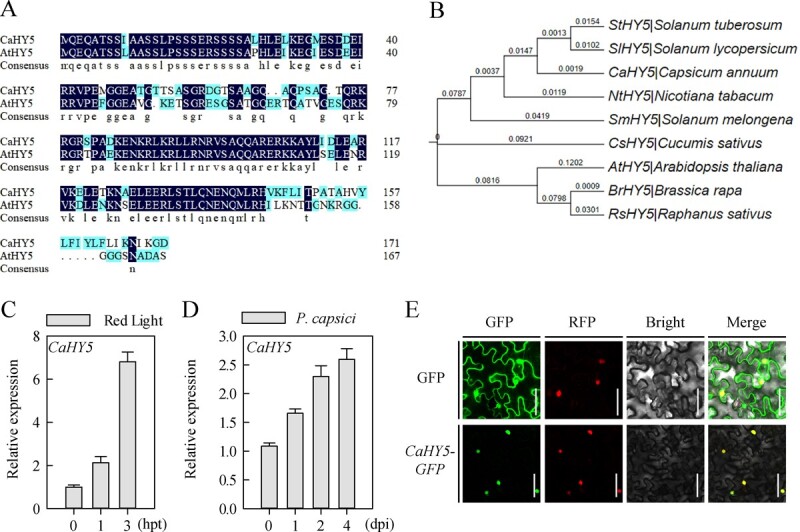
Red light and *Phytophthora capsici* induce the expression of *CaHY5* in pepper. **A** Analysis of deduced amino acid sequences of CaHY5. Alignment of deduced amino acid sequences of pepper CaHY5 (CA03g10440) and *Arabidopsis* AtHY5 (AT5G11260). **B** Phylogenetic tree of the CaHY5 homolog proteins. Phylogenetic tree of pepper CaHY5 (*Capsicum annuum*, CA03g10440) with representative related proteins tomato SlHY5 (*Solanum lycopersicum*, NM_001247891), potato StHY5 (*Solanum tuberosum*, XP_006361723), tobacco NtHY5 (*Nicotiana tabacum*, XP_016452267), eggplant SmHY5 (*Solanum melongena*, *NP_001234820*), cucumber CsHY5 (*Cucumis sativus*, XP_004138731), *Arabidopsis* AtHY5 (*Arabidopsis thaliana*, AT5G11260), rape BrHY5 (*Brassica rapa*, XP_009121971), and radish RsHY5 (*Raphanus sativus*, XP_018445811). **C** and **D** Expression levels of the *CaHY5* in pepper leaves analysed at the indicated time points after exposure of plants to red light (**C**) and infection with *P. capsici* (**D**), hours post infection (hpt). The relative *CaHY5* expression in treated plants was compared with control plants. Data represent the mean ± SE (*n* = 3). **E** CaHY5-GFP fusion protein localization in *N. benthamiana* leaves. H2B-RFP (RFP, red fluorescent protein) protein is a nuclear protein marker. Scale bars represent 50 μm.

HY5 acts downstream of the photoreceptor phytochrome B, and plays critical roles in plant growth, stress response, and several hormonal pathways in a light-dependent manner [12, 13, 24]. Therefore, the CaHY5 [25] protein was assumed to be an important candidate to further investigate the mechanism of action of red light in regulating pepper resistance to PCI. An alignment of deduced amino acid sequences of CaHY5 and its homolog in *Arabidopsis* (HY5) indicated that pepper CaHY5 has a high sequence similarity to AtHY5 (Fig. 2A). In addition, to investigate the relationship among CaHY5 and its orthologs in tomato (SlHY5), potato (StHY5), tobacco (NtHY5), eggplant (SmHY5), cucumber (CsHY5), *Arabidopsis* (AtHY5), rape (BrHY5), and radish (RsHY5), a phylogenetic analysis was conducted. As a result, CaHY5 showed the highest sequence similarity to SlHY5 (Fig. 2B). Next, RT­qPCR assays revealed that the application of red light and PCI significantly increased the expression of *CaHY5* in pepper plants (Fig. 2C and D). Moreover, as subcellular localization is associated closely with the potential molecular function of a TF, we examined the CaHY5 subcellular localization in *Nicotiana benthamiana* leaves by expressing the CaHY5-green fluorescent protein (GFP) fusion protein. The CaHY5-GFP fusion protein was found to be exclusively localized to cell nuclei (Fig. 2E), indicating that CaHY5 is localized in the nucleus.

**Figure 3:**
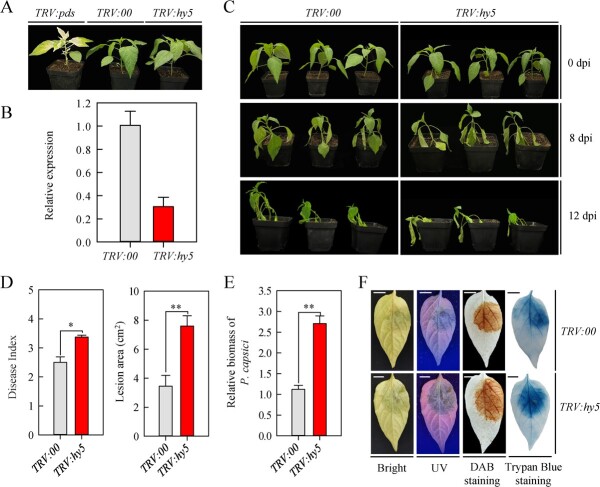
CaHY5 positively regulates pepper resistance to *Phytophthora capsici* infection. **A** Lines of *CaHY5*-silenced tobacco rattle virus (*TRV:00* and *TRV:hy5*) pepper plants generated by virus-induced gene silencing (VIGS). The *phytoene desaturase* (*CaPDS*)­silenced pepper plants (*TRV:pds*) exhibited a photo-bleaching phenotype as an indicator. **B** RT-qPCR analysis showing that the *CaHY5* expression was down-regulated in *CaHY5*-silenced pepper plants. **C** Resistance levels in *TRV:hy5* and *TRV:00* (empty vector control) pepper plants at 0, 8, and 12 dpi with *P. capsici*. **D** and **E** Disease index (8 dpi), lesion area (4 dpi), and *P. capsici* biomass (4 dpi) in *TRV:hy5* and *TRV:00* pepper plants*.* Data represent the mean ± SE (*n* = 3). **F** Increase in H_2_O_2_ levels and cell death in *TRV:hy5* pepper leaves at 4 dpi. Scale bar represents 1 cm.

Furthermore, to verify the role of *CaHY5* in pepper resistance to PCI, we generated *CaHY5*-silenced pepper plants via VIGS with the *TRV:hy5* construct, in which the expression of *CaHY5* was reduced (Fig. 3A and B). The *phytoene desaturase* (*CaPDS*)­silenced pepper plants (*TRV:pds*) exhibited a photobleaching phenotype, indicating successful silencing of the target gene. Compared with the control plants (infected with the *TRV:00* empty vector), the *CaHY5*-silenced pepper plants showed higher PCI susceptibility at 8 and 12 dpi (Fig. 3C), as indicated by significantly higher disease index, larger lesion area (Fig. 3D), and higher biomass of *P. capsici* (Fig. 3E), as well as increases in H_2_O_2_ accumulation and cell death (Fig. 3F). Overall, these data suggested that *CaHY5* positively regulates pepper resistance to PCI.

### CaHY5 enhances pepper resistance to PCI by directly activating the transcription of SA biosynthesis-related genes *CaPAL3* and *CaPAL7*

**Figure 4:**
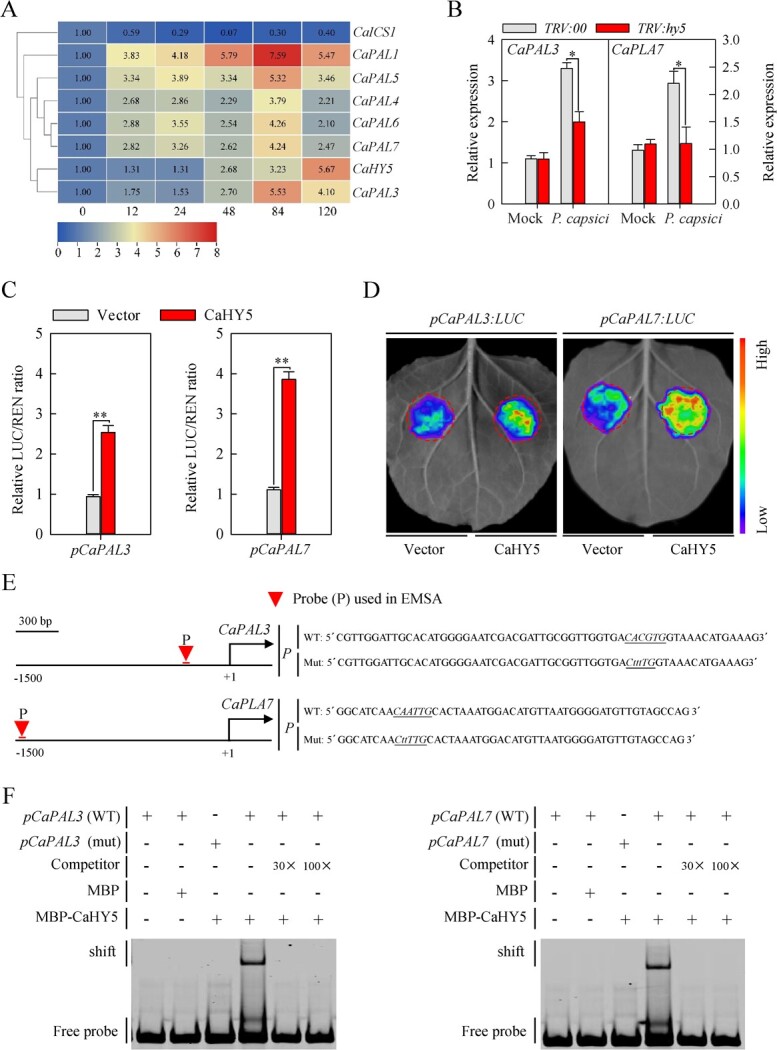
CaHY5 directly activates *CaPAL3* and *CaPAL7* expression. **A** Heatmap showing the expression patterns of *CaHY5* and SA biosynthesis­related *CaICS1* and *CaPALs* genes in pepper at 0, 12, 24, 48, 84 and 120 hours post inoculation (hpi) with *Phytophthora* pathogen. The log_2_FC (fold change) was indicated using the color bar. **B** Expression levels of *CaPAL3* and *CaPAL7* were analysed in *TRV:hy5* and *TRV:00* pepper leaves at 4 dpi with *P. capsici*. **C** CaHY5 transcriptionally activates the reporter gene of *LUC* (driven by the *CaPAL3* and *CaPAL7* promoters) by a transient expression assay. **D** Dual-luciferase reporter assay shows that CaHY5 activates the *LUC* reporter gene expression driven by *CaPAL3* and *CaPAL7* promoters. The LUC/REN ratio indicates the relative activity of *CaPAL3* and *CaPAL7* promoters. Three independent transient expression experiments were performed. **E** Schematic diagrams of *CaPAL3* and *CaPAL7* promoter sequence selection for the EMSA. The triangles indicate EMSA sequence position. **F** EMSA shows that CaHY5 directly binds to the *CaPAL3* and *CaPAL7* promoters. Data represent the mean ± SE (n= 3).

To investigate the possible mechanism by which CaHY5 modulates the PCI resistance of pepper, we attempted to identify the genes targeted by CaHY5 and involved in SA biosynthesis. Previous studies have demonstrated that two independent pathways for synthesizing SA from chorismate, including phenylalanine ammonia-lyase (PAL) [26, 27] and isochorismate synthase (ICS) [28]. We first evaluated the expression profile of *CaHY5*, *CaPALs*, and *ICS1* genes in pepper leaves under *Phytophthora* pathogen infection by analysing previously published RNA­sequencing data [29]. The results showed that pathogen infection induced the dynamic expression of *CaHY5* and *CaPALs* (Fig. 4A). Next, we determined the expression of *CaICS1* and *CaPALs* in control plants and *CaHY5*­silenced pepper plants under PCI. As a result, the *CaHY5*-silenced pepper plants showed significantly lower expression of *CaPAL3*, *CaPAL6*, and *CaPAL7* under PCI compared with the control plants (Fig. 4B; Fig. S5A, see online supplementary material). By contrast, other genes (*CaPAL1*, *CaPAL2*, *CaPAL4*, *CaPAL5*, and *CaISC1*) showed no significant reduction of expression (Fig. S5A and B, see online supplementary material). Moreover, we identified the G-box and E-box, which may be the binding motif by the HY5 TF in the *CaPAL3* and *CaPAL7* promoters (Fig. S5C, see online supplementary material), implying that these two genes are directly regulated by CaHY5. To test this result, we first examined the effect of CaHY5 on *CaPAL3* and *CaPAL7* expression through dual-luciferase reporter assays in *N. benthamiana* leaves (Fig. S5D, see online supplementary material). The results showed that *CaHY5* driven by the constitutive *CaMV35S* promoter co-expressed with *LUC* driven by the *CaPAL3* and *CaPAL7* promoters increased *LUC* expression and LUC luminescence intensity compared with the empty vector control (Fig. 4C and D).

**Figure 5:**
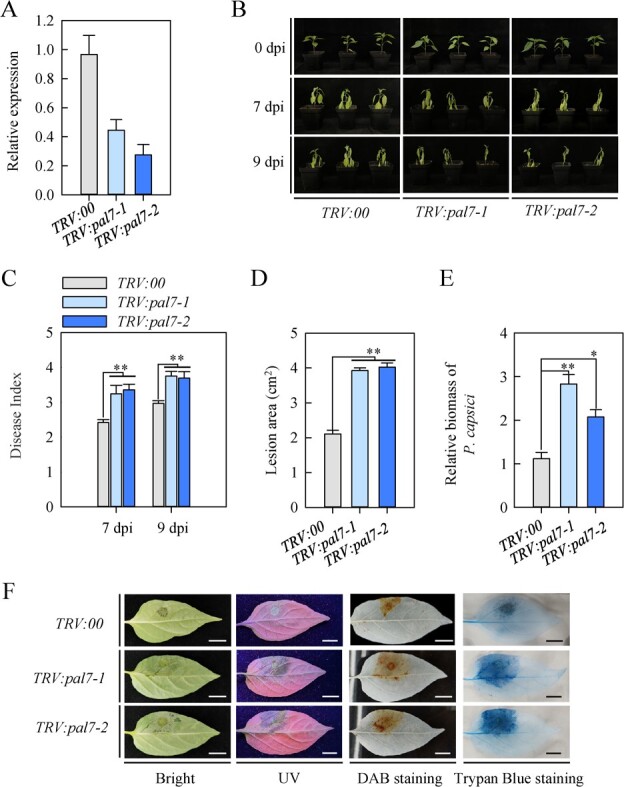
*CaPAL7* silencing increases PCI susceptibility in pepper plants. **A** Analysis of RT-qPCR data reveals that the *CaPAL7* expression in *CaPAL7*­silenced pepper plants is down-regulated. **B** Resistance level in *TRV:pal7* and *TRV:00* (empty vector control) pepper plants at 9 dpi with *P. capsici*. **C**–**E** Disease index (**C**, 7 and 9 dpi), lesion area (**D**, 4 dpi), and *P. capsici* biomass (**E**, 4 dpi) in *TRV:pal7* and *TRV:00* during PCI in pepper*.* Data represent the mean ± SE (*n* = 3). **F** Increase in H_2_O_2_ levels and cell death in *TRV:pal7* pepper leaves at 4 dpi. Scale bar represents 1 cm.

Next, we performed an electrophoretic mobility shift assay (EMSA) with maltose­binding protein (MBP)-CaHY5 fusion and DNA probes designed from the *CaPAL3* and *CaPAL7* promoters harboring the G-box (CACGTG) and E-box (CAATTG), and we mutated the G-box and E-box to CtttTGG and CttTTG, respectively. The results revealed that MBP-CaHY5, but not MBP alone, directly bound to DNA probes (WT), followed by repression of the competitors. Meanwhile, the binding of MBP-CaHY5 fusion protein and DNA probes were totally abolished when the G-box and E-box were mutated (Fig. 4E and F). Moreover, we generated *CaPAL3*­ and *CaPAL7*­ silenced pepper plants through VIGS, in which the expression of *CaPAL3* and *CaPAL7* were reduced (Fig. 5A; Fig. S6A, see online supplementary material). Compared with the control plants, the *CaPAL3*­ and *CaPAL7*-silenced pepper plants showed higher PCI susceptibility at 9 dpi (Fig. 5B; Fig. S6B, see online supplementary material), as indicated by significantly higher disease index (Fig. 5C; Fig. S6C, see online supplementary material), larger lesion area (Fig. 5D; Fig. S6D, see online supplementary material ), and higher biomass of *P. capsici* (Fig. 5E; Fig. S6E, see online supplementary material ), as well as increases in H_2_O_2_ accumulation and cell death (Fig. 5F; Fig. S6F, see online supplementary material), indicating *CaPAL3* and *CaPAL7* act positively on pepper immunity to PCI. Collectively, these results demonstrated that CaHY5 positively modulates pepper resistance to PCI by directly activating the expression of *CaPAL3* and *CaPAL7*.

### Exogenous application of SA rescues the resistance of *CaHY5*-silenced pepper plants to PCI

**Figure 6:**
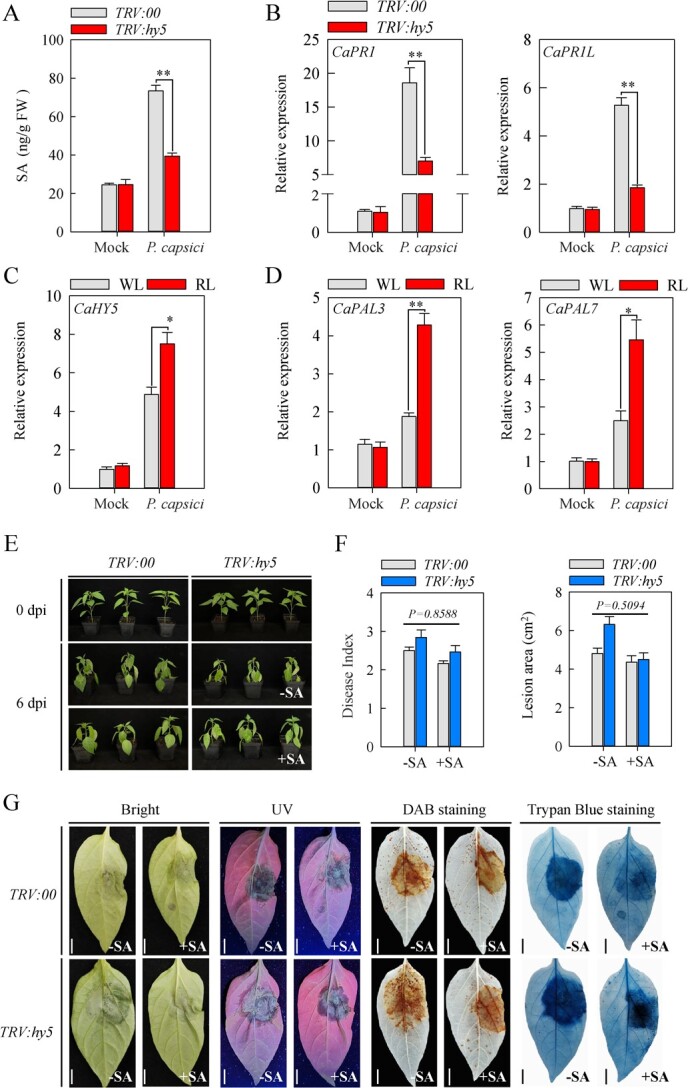
Application of exogenous SA restores resistance levels of *TRV:hy5* pepper plants to PCI. **A** Contents of endogenous SA were measured in pepper leaves at 4 dpi with *P. capsici*. **B** Expression levels of SA-responsive *CaPR1* and *CaPR1L* analysed by RT-qPCR in *TRV:hy5* and *TRV:00* pepper leaves at 4 dpi with *P. capsici*. **C** and **D** The expression levels of *CaHY5*, *CaPAL3*, and *CaPAL7* genes were up-regulated by exposure of pepper plants to red light during PCI at 4 dpi. **E** Resistance analysis of *TRV:hy5* and *TRV:00* pepper plants to PCI upon exogenous application of SA (1 mM). Photographs were acquired at 0 and 6 dpi. **F** Disease index and lesion area in *TRV:hy5* and *TRV:00* pepper plants to PCI were decreased by exogenous SA (1 mM) treatment. **G** H_2_O_2_ levels and cell death in *TRV:hy5* and *TRV:00* pepper plants to PCI were decreased by exogenous SA (1 mM) treatment. Scale bars represent 1 cm. Data represent the mean ± SE (*n* = 3).

To further determine whether CaHY5 is involved in the SA-mediated resistance of pepper to PCI, we first detected the SA content in *CaHY5*-silenced and empty-vector TRV pepper plants at 4 dpi. *CaHY5*-silenced pepper plants showed significantly lower PCI-induced SA accumulation than the TRV plants, indicating that *CaHY5* positively regulates SA accumulation in pepper under PCI (Fig. 6A). Next, we performed RT­qPCR analysis on the TRV and *CaHY5*-silenced pepper plants under PCI. The results demonstrated that *CaHY5*-silenced pepper plants had significantly lower PCI-induced expression of SA­responsive genes *CaPR1* and *CaPR1L* compared with the TRV plants (Fig. 6B). Consistently, PCI significantly increased the expression of *CaHY5*, *CaPAL3*, and *CaPAL7* in red light-treated pepper plants as compared to white light-treated plants (Fig. 6C and D).

To further validate the role of *CaHY5* in regulating SA-mediated PCI resistance of pepper, we compared the effect of exogenous SA application on *CaHY5*-silenced and control pepper plants in terms of PCI susceptibility. As expected, both *CaHY5*-silenced and control pepper plants displayed certain disease symptoms of PCI, with more serious symptoms being observed in *CaHY5*-silenced plants relative to the control plants. In contrast, *CaHY5*­silenced plants treated with exogenous SA displayed similar PCI susceptibility to the control plants without exogenous SA treatment (Fig. 6E; Fig. S7, see online supplementary material), as evidenced by similar disease index and lesion area (Fig. 6F). In addition, DAB and trypan blue staining assays further revealed that exogenous SA treatment repressed PCI-induced H_2_O_2_ accumulation and cell death in *CaHY5*-silenced pepper plants, which were close to the levels in the control plants without exogenous SA application (Fig. 6G), indicating that exogenous SA treatment could rescue the resistance of *CaHY5*­silenced pepper plants to PCI. Similarly, *Caphyb2*­silenced pepper plants treated with exogenous SA displayed similar susceptibility to PCI as the control plants without exogenous SA treatment (Fig. S8A, see online supplementary material), as indicated by the similar disease index and lesion area (Fig. S8B and C, see online supplementary material). Collectively, these results confirmed that *CaHY5* positively regulates pepper resistance to PCI by promoting SA accumulation and SA­mediated immunity.

## Discussion

**Figure 7:**
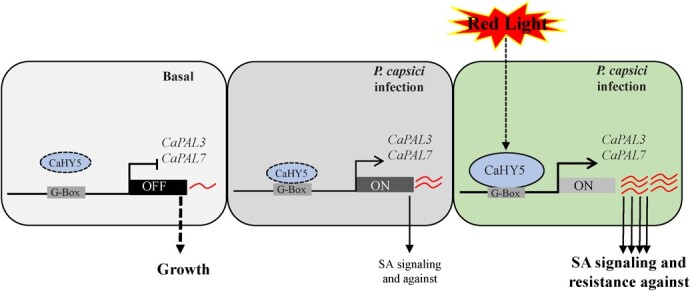
A working model for red light signals-induced activation of CaHY5 and subsequent enhancement of pepper resistance to *Phytophthora capsici* infection (PCI) by regulating SA-mediated immunity.

Production of peppers is severely restricted by Phytophthora blight, the most destructive soil-borne disease caused by *P. capsici*. Greenhouse cultivation is the most common and effective method of vegetable crops (such as pepper, tomato, and eggplant) production worldwide. However, plastic films covering the greenhouse reduce the incoming light intensity, resulting in reduced plant growth and pathogen defense. Moreover, higher humidity in the greenhouse favors the growth of all stages of *P. capsici* [30]. This study revealed that red light enhances pepper resistance to PCI by inducing SA accumulation and the expression of SA-responsive genes *CaPR1* and *CaPR1L*. Our results further demonstrated that the application of red light can up­regulate the expression of *CaHY5* TF, which then activates the expression of *CaPAL3* and *CaPAL7* by directly binding to their promoters, thereby enhancing SA­mediated resistance of pepper to PCI (Fig. 7). This study reveals a new mechanism linking red light signaling to SA accumulation in pepper under PCI and demonstrates that *CaHY5* plays a critical role in regulating light signal-induced SA biosynthesis.

In uninfected pepper plants, the low expression level of SA biosynthesis-related genes and SA contents maintain plant growth. In infected pepper plants, basal SA-mediated immunity is initiated in response to PCI. By contrast, the CaHY5 positively and directly regulates the expression of SA biosynthesis-related *CaPAL3* and *CaPAL7* genes, as well as upregulates the expression of SA­responsive *CaPR1* and *CaPR1L* genes, thereby conferring enhanced SA-mediated immunity and resistance against PCI. Taken together, red light signals induce SA accumulation by activating CaHY5 to enhance pepper resistance to PCI.

### Red light is a critical environmental signal for plant immunity against pathogens

As a critical external signal, light regulates plant immunity to the attack of various pathogens [1, 2, 31]. For instance, compared with light conditions, dark conditions resulted in more serious disease symptoms in *Arabidopsis* during pathogen infection, accompanied by decreases in SA accumulation and *PR1* expression [32]. It is generally believed that compared with other types of light, red light is a more effective external factor to enhance plant immunity [4, 33]. Here, we provide several lines of evidence that red light enhances pepper resistance to PCI depending on SA accumulation and SA­mediated immunity. First, compared with white light, red light could significantly reduce the disease index and lesion area of pepper plants exposed to PCI (Fig. 1B). Second, SA measurement and RT-qPCR assays revealed that red light treatment increased the SA content in pepper plants compared with white light treatment (Fig. 1E), accompanied by upregulated expression of *CaPR1* and *CaPR1L*, two SA­responsive genes (Fig. 1F). Third, PCI assays with *TRV:00* and *TRV:phyb2* pepper plants indicated that the red light photoreceptor *CaPHYB2* plays a positive role in regulating PCI resistance (Fig. S4, see online supplementary material). Notably, red light-absorbing photoreceptors, phytochromes have been identified to play critical roles in regulating plant responses to stresses [34]. Collectively, red light represents an external factor or signal that induces resistance to PCI through phytochrome (CaPHYB) and SA-mediated immunity in pepper. Moreover, the application of red light to horticultural crops is an environment-friendly and innovative method to enhance crop resistance to pathogen attack [4].

### CaHY5 positively regulates pepper resistance to PCI by modulating SA­mediated immunity

As plants are sessile organisms, it is critical to precisely allocate the limited available resources between growth and defense response under unfavorable environmental conditions. Thus, plants respond to unfavorable factors in their immediate environment by activating signal transduction pathways and gene transcriptional reprogramming, which depend on extracellular and subcellular information of TFs [35, 36]. In this regard, HY5, a typical bZIP TF acting downstream of multiple photoreceptors including phytochromes, is crucial for the light signaling pathway [12, 37]. Here, we demonstrated that CaHY5 positively regulates pepper resistance to PCI. On the one hand, the application of red light and PCI induced the expression of *CaHY5* (Fig. 2C and D). In addition, *CaHY5*-silenced pepper plants exhibited more severe disease symptoms and larger lesion areas than the control plants (Fig. 3C and D). On the other hand, SA measurement and RT-qPCR assays showed that *TRV:hy5* pepper plants had a lower level of SA content than the control plants (*TRV:00*), and also down-regulated expression levels of *CaPAL3*, *CaPAL7*, *CaPR1*, and *CaPR1L* (Figs 4B and 6B)*.* Further relative LUC/REN ratio analysis and EMSA revealed that *CaHY5* activates the expression of *CaPAL3* and *CaPAL7* by directly binding to their promoters (Fig. 4C and F). Notably, the PAL pathway is one of the rate-limiting steps in SA biosynthesis, which leads to SA accumulation and promotes SA­mediated immunity [26, 38].

### Red light enhances pepper resistance to PCI through CaHY5-regulated gene expression

Previous studies have shown that SA plays a critical role in plant defense against biotrophic and hemibiotrophic pathogens during PTI or ETI [38, 39]. Exogenous SA or its biologically active analogs can enhance resistance against biotrophic and hemibiotrophic pathogens [40]. Transgenic plants overexpressing the SA hydroxylase *NahG*, which degrades SA into catechol, showed a significant reduction in SA accumulation and enhanced susceptibility to pathogens in *Arabidopsis* and tobacco [41, 42]. Here, our results demonstrated that exogenous application of SA could enhance pepper resistance to PCI (Fig. S1, see online supplementary material), indicating that SA plays a positive role in pepper resistance to PCI. Red light induces SA accumulation and the expression of SA-responsive genes during PCI in pepper, and the expression of *CaHY5* depends on red light. Moreover, the expression pattern of *CaHY5* was correlated with the changes in the expression of SA biosynthesis-related genes *CaPAL3* and *CaPAL7* (Fig. 4A). Importantly, our further analysis of *TRV:00* and *TRV:hy5* pepper plants revealed that exogenous SA treatment could restore the susceptibility of *TRV:hy5* pepper plants to PCI (Fig. 6E and F). Consistently, in previous studies, several TFs were found to play critical roles in SA accumulation by regulating the expression of SA biosynthesis-related genes [43, 44]. StbZIP61 regulates SA biosynthesis by directly activating the expression of *StICS1*, a key enzyme for SA synthesis, which is essential for SA biosynthesis, thereby promoting SA-mediated immunity of potato to *Phytophthora infestans* infection [43]. Taken together, the discovery of genes and transcription factors that enhance pepper resistance to PCI is of great significance to developing resistant pepper varieties, thereby preventing the outbreak of Phytophthora blight. Our results provide novel insights into the molecular mechanism underlying the resistance of pepper to PCI, and CaHY5 was identified as a potential candidate gene for the improvement of PCI resistance in pepper. In addition, these findings suggest that LED-derived red light can be exploited to further improve the resistance of horticultural crops to major diseases including Phytophthora blight through the manipulation of light quality in greenhouse cultivation.

## Materials and methods

### Plant materials and growth conditions

Seeds of pepper (*C. annuum* L.) Hangjiao12 and *N. benthamiana* were used for all experiments in this study. All pepper seeds were placed on moistened sterile filter paper in Petri dishes for 72–96 h at 28 ± 2°C for germination, and then sown in a plastic tray filled with soil mixture (peat moss and vermiculite mixed at 2: 1, v/v). Pepper and *N. benthamiana* plants were grown at 25°C light/20°C ± 2°C dark under a 12­light/12­dark cycle with a photosynthetic photon flux density (PPFD) of 200 μmol photons m^−2^ s^−1^ in a growth room. At the age of 6 weeks, the *N. benthamiana* young leaves where fully expanded were used for dual-luciferase reporter assays and subcellular localization.

Pepper plants were exposed to red light with a maximum wavelength of 660 nm using light-emitting diodes (LEDs, Huizhou Kedao Technology Co. Ltd, Huizhou, China) light at the 6-leaf stage, and those under white LED light were set as the control. Light intensity at canopy level was set at 200 μmol m^−2^ s^−1^ PPFD for red and white light.

### *P. capsici* infection assays

The *P. capsici* isolate JX202105 was cultured on potato dextrose agar (PDA) medium in the dark for 8 d at 25°C. Then, the mycelia were scraped from the plate, and incubated under the light at 25°C until the formation of sporangia. For zoospore preparation, sterile double distilled water was added, followed by further incubation for 40 min at 4°C.

For *P. capsici* infection assay in pepper roots. The pepper plants grown in pots till the 6-leaf stage, and the pots were infected through adding the 3 mL suspension of zoospores (approximately 1 × 10^5^ spores per mL), then were transferred to the same growth chamber for the pathogenicity test. Using the injury index of disease as described previously [45], we calculated the resistance levels of pepper plants to *P. capsici* infection. For the *P. capsici* infection in leaves, the pepper plants grown in pots till the 6-leaf stage, leaves were excised. The 20 μL droplets of zoospore suspension were inoculated onto the abaxial surface center of detached leaves, and the leaves were maintained at 25°C for 4 d. Plants inoculated with ddH_2_O were used as Mock. Subsequently, the disease development was recorded and photographed, and the disease area and total leaf area of each image were analysed through *Image­Pro Plus 6.0* image software.

### Molecular cloning and plasmid construction

DNA constructs for all experiments were generated following standard molecular biology protocols and using ClonExpress Ultra One Step Cloning (Vazyme) technology. For subcellular localization assay, pepper cDNA was used as a template for PCR amplification of *CaHY5* coding sequence (CDS) and cloned into the *Xba*I*­Xma*I sites of the *pSuper1300-GFP* vector to generate the *pSuper-CaHY5-GFP* construct. For the VIGS assay, the specific fragments of *CaPHYB2*, *CaHY5*, *CaPAL3*, and *CaPAL7* were amplified by PCR using pepper cDNA as templates, and cloned into the *EcoR*I*­Xho*I sites of the *pTRV2* vector to generate the *pTRV2­phyb2*, *pTRV2-hy5*, *pTRV2-pal3*, and *pTRV2-pal7* constructs. For dual-luciferase reporter assay, the promoter fragments of *CaPAL3* and *CaPAL7* were amplified by PCR using pepper genomic DNA as template, and cloned into the *Hind*III­*BamH*I sites of the *pGreen*­*0800*­*LUC* vector to generate the *pCaPAL3:LUC* and *pCaPAL7:LUC* reporter constructs. In addition, a fragment of *CaHY5* CDS was cloned into the *BamH*I­*Hind*III sites of the *pGreenII62*­*SK* vector to generate the *35S*­*CaHY5*­*SK* effector construct driven by the *CaMV35S* promoter. For the electrophoretic mobility shift assay, a fragment of *CaHY5* CDS was cloned into the *EcoR*I*­Sal*I sites of the *pMAL*­*c4X* vector to generate the *pMBP-CaHY5* construct. Primers used for generating DNA constructs are listed in Table S2 (see online supplementary material).

### Virus-induced gene silencing (VIGS)

For VIGS assays [46]. In brief, the specific fragments of *CaHY*5, *CaPHYB2*, *CaPAL3*, and *CaPAL7* were identified by searching via the VIGS tool (https://vigs.solgenomics.net/) and BLAST analysis against the pepper genome sequences. The *pTRV1*, *pTRV2*, *pTRV2­pds*, *pTRV2­hy5*, *pTRV2­phyb2*, *pTRV2­pal3*, and *pTRV2­pal7* plasmids were transformed into *Agrobacterium tumefaciens* (GV3101), respectively. Using a needleless syringe, different combinations of *Agrobacterium* were co-infiltrated into 3­week­old pepper leaves (two cotyledons and one true leaf). Then, the pepper seedlings were kept in a growth incubator under dark conditions at 18°C for 56–60 h. After that, seedlings were grown in a growth room for 30 d before being used for the experiments.

### Subcellular localization

For subcellular localization of CaHY5, the *A. tumefaciens* harbouring the *pSuper­CaHY5­GFP* (or *pSuper1300-GFP*) and a nuclear marker protein (*pCaMV35S-H2B-RFP*) [47] were co-infiltrated into the *N. benthamiana* leaves. After 36–48 h, fluorescence signals from GFP or red fluorescent protein (RFP) were analysed using a confocal laser­scanning microscope (Nikon C2-ER, Tokyo, Japan).

### Phylogenetic analysis

In order to perform phylogenetic analysis, MEGA6 software was used to align full-length amino acid sequences and construct phylogenetic trees. The consensus Neighbor-joining tree was obtained from amino acid sequences with a bootstrap value of 1000 replications. In addition, the percentage at branch points represents the posterior probabilities of sequences.

### Electrophoretic mobility shift assay (EMSA)

For EMSA, *Escherichia coli* BL21 cells were transformed with recombinant *MBP-CaHY5* and empty vector constructs, then the MBP*­*CaHY5 fusion protein was induced with IPTG (500 μM) in LB medium for 16–20 h. Then, the recombinant MBP fusion proteins were purified on Amylose Resin (NEB) and quantified through the bovine serum albumin (BSA). To generate Cy5*­*labeled probes, the fragments of *CaPAL3* and *CaPAL7* promoters were synthesized (sequences listed in Supplementary Table S2, see online supplementary material). As indicated, the probes were incubated in 20 μL reaction mixtures with MBP*­*CaHY5 or MBP (2 μg) for 30 min at 25°C. Subsequently, using 12% native polyacrylamide gels, the samples were separated, and the fluorescent labels were detected by a LI*­*COR Odyssey Infrared Imaging System (Odyssey, Lincoln, NE, USA).

### Transient transcription dual-luciferase reporter assay

For the dual-luciferase reporter (DLR) assay [48]*.* In brief, the effector plasmids (*35S*­*CaHY5*­*SK* and empty vector) and reporter plasmids (*pCaPAL3:LUC* and *pCaPAL7:LUC*) were transformed into *Agrobacterium strain* EHA105 (*pSoup*). Different combinations of bacteria were co-infiltrated into leaves of *N. benthamiana* plants (6­week­old) using a needleless syringe. After 36–48 h, the leaves were infiltrated with 1 mM luciferin (number 88294, Pierce, Thermo Scientific, USA), and the firefly LUC signal was detected by Night SHADE LB 985 system (Berthold, Germany). According to the manufacturer’s instructions, firefly LUC and the control Renilla LUC activities of the co-infected leaves were measured using the Dual-Glo Luciferase Assay System (Promega, USA). Then, we calculated the relative LUC/REN ratio by normalizing the LUC activity to the REN activity driven by the *CaMV35S* promoter.

### Histochemical staining assay

For 3, 3′-diaminobenzidine (DAB, number D12384, Sigma-Aldrich) and trypan blue (T6146, Sigma-Aldrich) staining [49], the detached youngest leaves of pepper plants under different treatments were infected with *P. capsici*. Then, leaves were collected after 3 d of infection, and stained with DAB and trypan blue solution. For DAB staining, the leaves were incubated in DAB (pH 3.8; 1 mg mL^−1^) solution for 6 h at 25°C in the dark. Subsequently, we cleared the leaves by boiling them in a lactic : glycerol : absolute ethanol (1 : 1 : 3 by volume) solution and further destained them in absolute ethanol. Trypan blue staining were performed by boiling the *P. capsici*-infected leaves for 2 min in the lactophenol-trypan blue staining solution [49], followed by an 8 h incubation at room temperature. Then, destaining was performed with chloral hydrate solution (2.5 g/mL).

### Free SA measurement

Pepper leaves (0.2 g) were ground with liquid nitrogen into powder, and incubated overnight in 1 mL of 90% methanol at 4°C. Then, the supernatants were collected after centrifuging the samples at 8000 *g* for 10 min. The precipitates were redissolved for 2 hours with 0.5 mL 90% methanol at 4°C, and supernatants were collected. The supernatants were then merged and dried under nitrogen, and the residues were dissolved in a 1 mL solution (ethyl acetate: cyclohexane, 1 : 1 by volume), and free SA was analysed using High-Performance Liquid Chromatography (HPLC, Waters 2695).

### Expression analysis

The relative expression profiles of genes were analysed in pepper with publicly available RNA sequencing data (number: SRP106410 and SRP119199) in the sequence read archive database (https://www.ncbi.nlm.nih.gov/sra) [32]. The gene expression levels were calculated using the TopHat-Cufflinks pipeline based on the Log_2_­transformed FPKM (FPKM, fragments per kilobase of exon per million fragments mapped) values. Subsequently, heatmap clustering of the *CaPAL* genes was performed according to Log_2_­transformed FPKM values with the TBtools software [50].

For gene expression analysis [47], in brief, we harvested pepper leaves and isolated total RNA with TRIzol reagent (Invitrogen). Then, the Bio­Rad iCycler iQTM Real-Time PCR Detection System was used to determine gene expression levels through ChamQ SYBR qPCR Master Mix (Vazyme) and gene-specific primers (sequences listed in Table S2, see online supplementary material). Pepper *Actin1* (*CaActin1*) was used as an internal control to quantify the relative expression levels.

### Statistical analysis

Statistically significant differences between groups were determined by Student’s *t*-test, with asterisks indicating significant differences (*P < 0.05 or **P < 0.01) in figures. In each experiment, at least three biological replicates were performed and analysed separately. All values are represented as means ± standard error (SE).

#### Accession numbers

This article includes sequence data that can be found on Genome Initiative (https://solgenomics.net/): *CaHY5* (CA03g10440, Capana08g000273); *CaPAL1* (CA10g12380, Capana00g003499); *CaPAL2*(CA12g15510, Capana03g003491); *CaPAL3* (CA05g20790, Capana05g002560); *CaPAL4* (CA00g95510, Capana09g002190); *CaPAL5* (CA09g02410, Capana09g002199); *CaPAL6* (CA09g02430, Capana09g002200); *CaPAL7* (CA09g02420, Capana11g001287); *CaPR1* (CA01g31060, Capana08g002192); *CaPR1L* (CA01g31110, Capana08g002192); and *CaActin* (CA12g08730, Capana12g001934).

## Supplementary Material

Supplementary_Figures_S1_uhad213

Supplementary_Figures_S2_uhad213

Supplementary_Figures_S3_uhad213

Supplementary_Figures_S4_uhad213

Supplementary_Figures_S5_uhad213

Supplementary_Figures_S6_uhad213

Supplementary_Figures_S7_uhad213

Supplementary_Figures_S8_uhad213

Supplementary_Tables_S1-2-HR-2023-625-10-9-F1_uhad213

Supplementary_Figures_S1-8-HR-2023-625-10-9-F1_uhad213

## Data Availability

All relevant data supporting our findings are available in the manuscript file，supplementary data, and publicly available RNA sequencing data (number: SRP106410 and SRP119199) in the National Center for Biotechnology Information (NCBI) Sequence Read Archive database (https://www.ncbi.nlm.nih.gov/sra).
