# Supplementary figures and images for: Red light induces salicylic acid accumulation by activating CaHY5 to enhance pepper resistance against Phytophthora capsici
Source: Hortic Res. 2023 Oct 17;10(11):uhad213. doi: 10.1093/hr/uhad213 (PMC10689078; doi:10.1093/hr/uhad213)

A

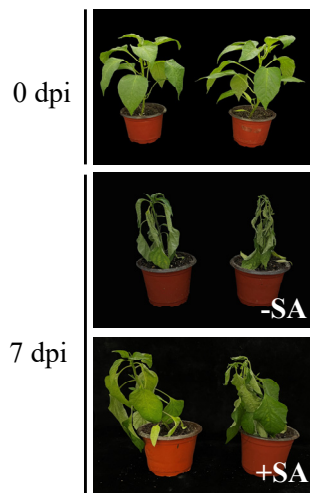

B

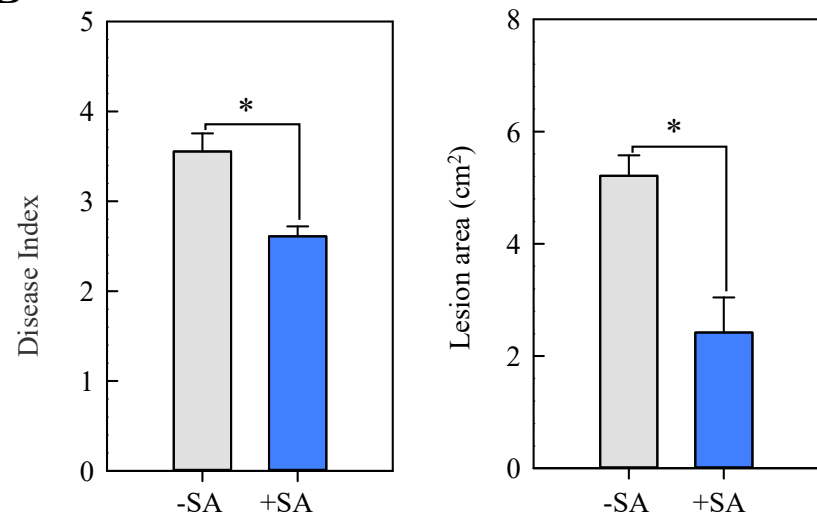

C

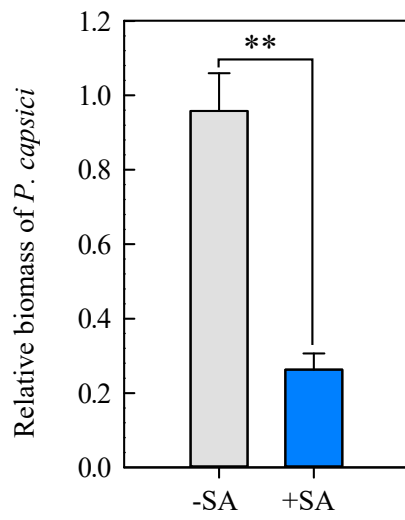

D

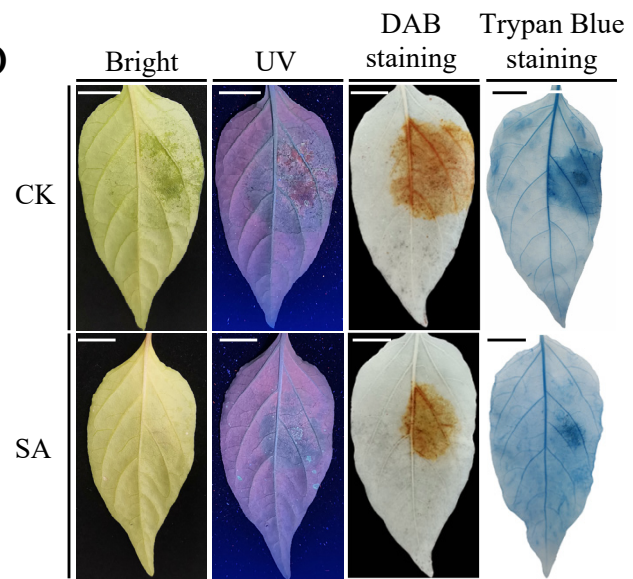

Supplement: Supplementary_Figures_S1_uhad213 [file supplementary_figures_s1_uhad213.pdf]

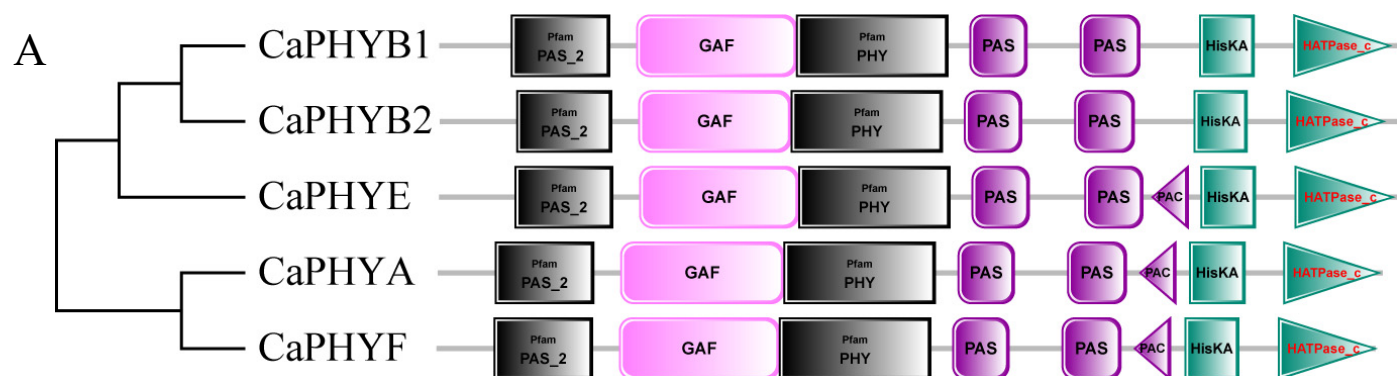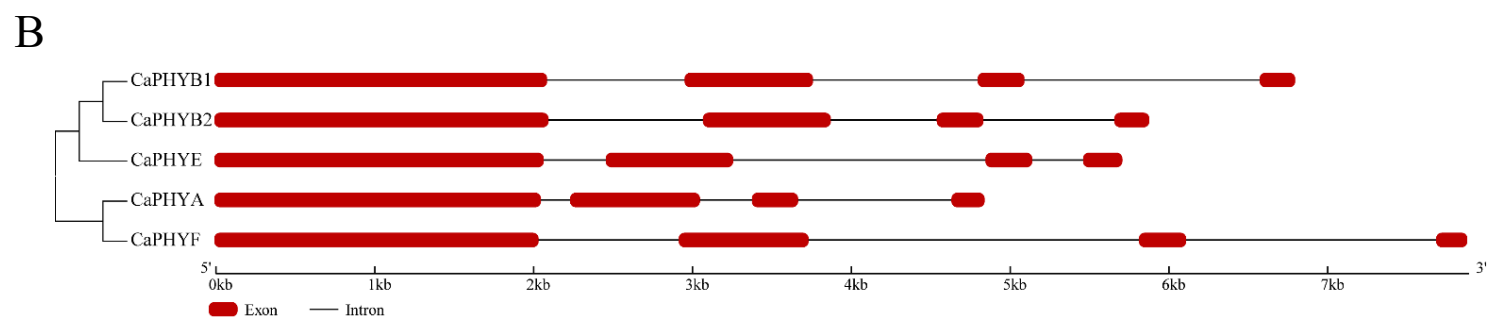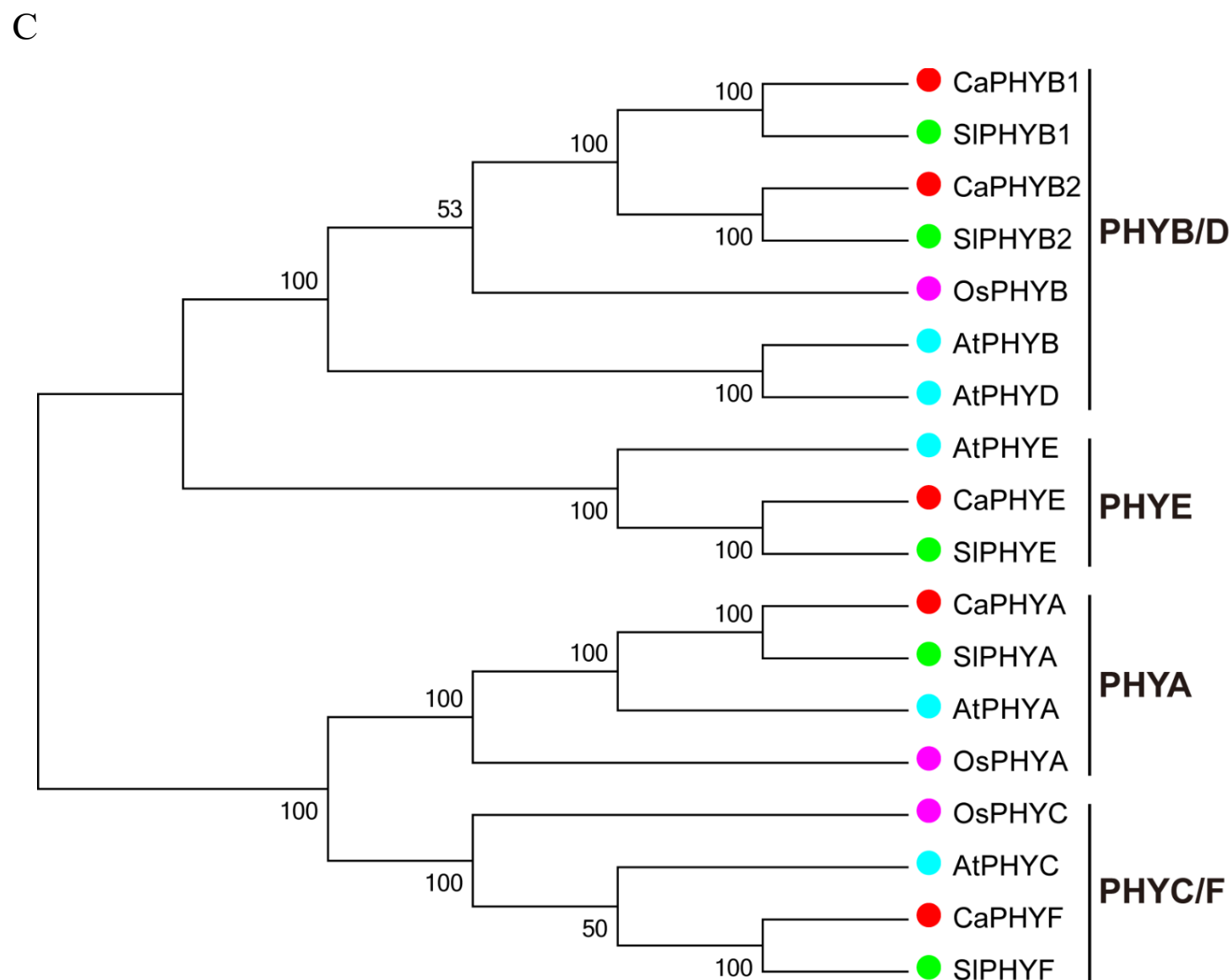

Supplement: Supplementary_Figures_S2_uhad213 [file supplementary_figures_s2_uhad213.pdf]

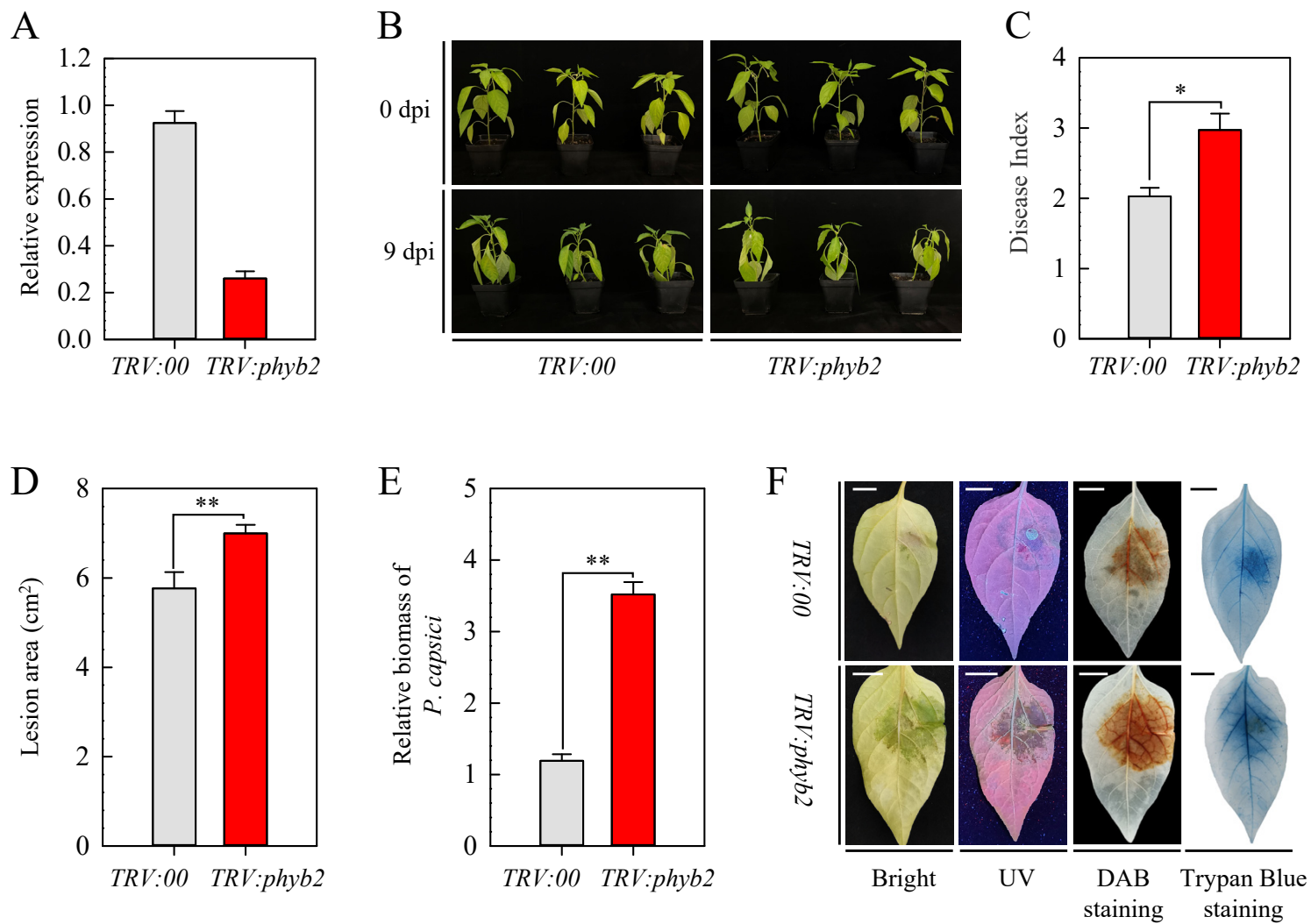

Supplement: Supplementary_Figures_S4_uhad213 [file supplementary_figures_s4_uhad213.pdf]

**A**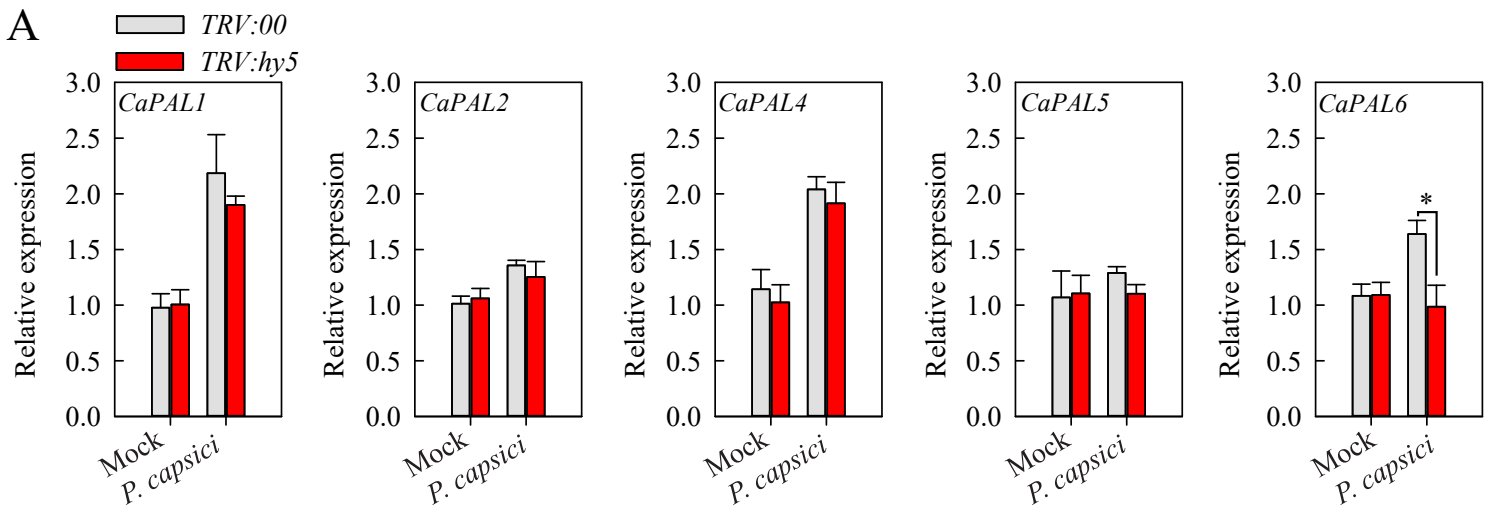**B**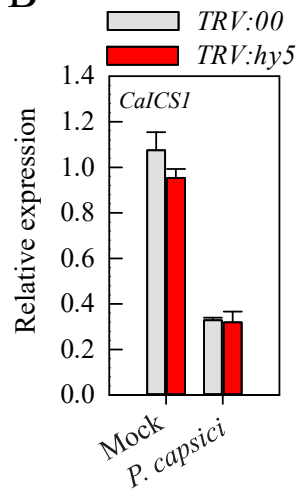**C**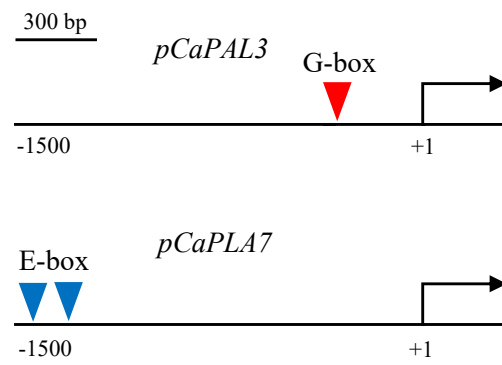**D**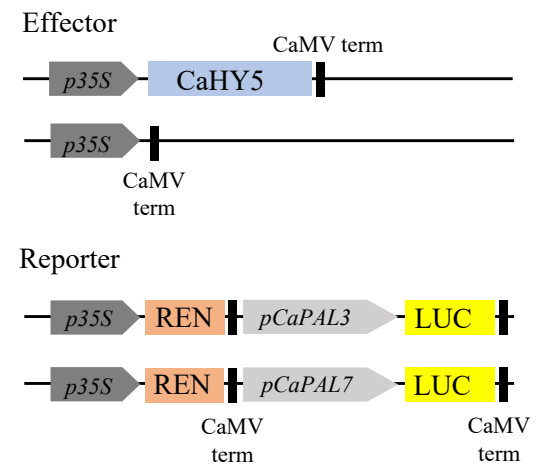

Supplement: Supplementary_Figures_S5_uhad213 [file supplementary_figures_s5_uhad213.pdf]

A

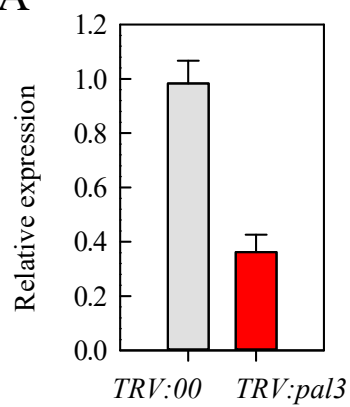

B

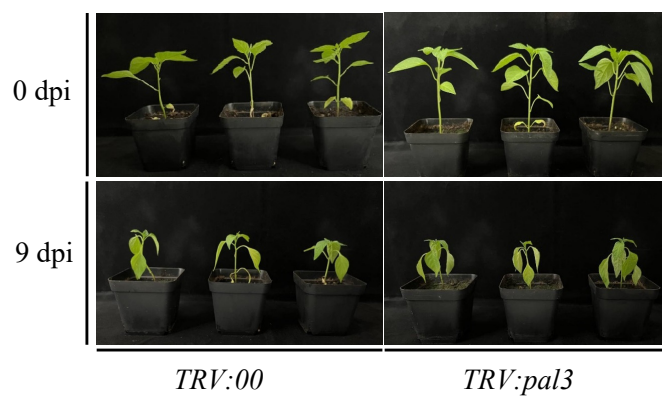

C

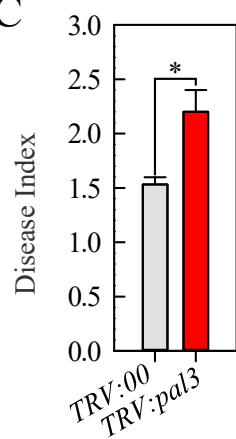

D

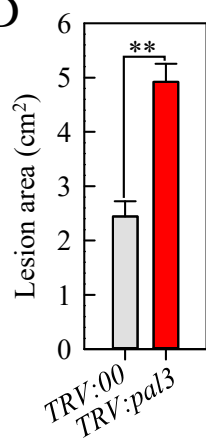

E

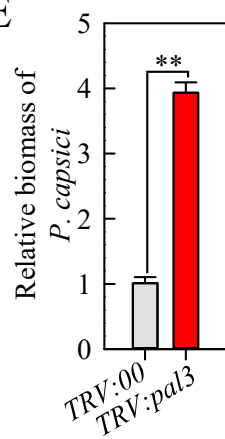

F

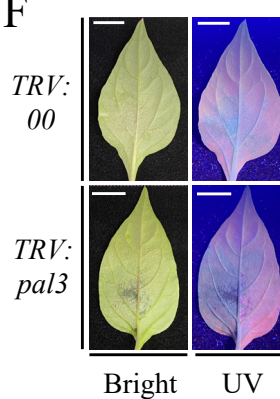

Supplement: Supplementary_Figures_S6_uhad213 [file supplementary_figures_s6_uhad213.pdf]

*TRV:00*

*TRV:hy5*

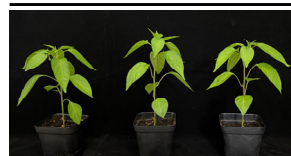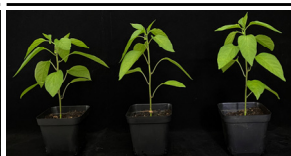

0 dpi

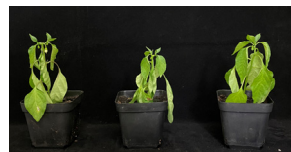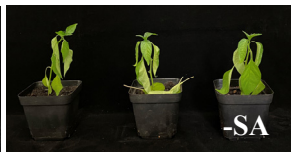

8 dpi

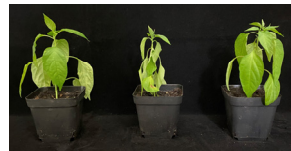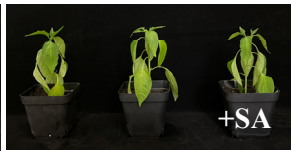

8 dpi

*TRV:00*

*TRV:hy5*

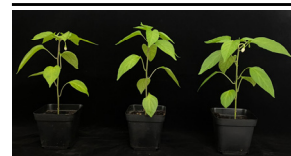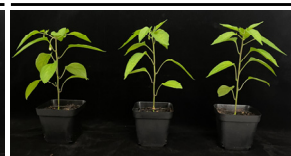

0 dpi

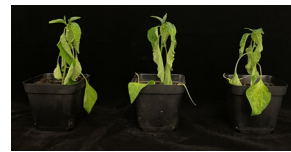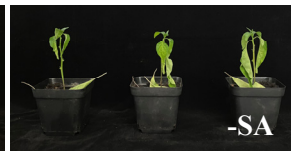

11 dpi

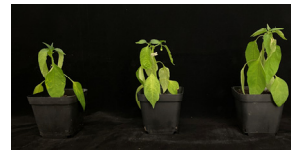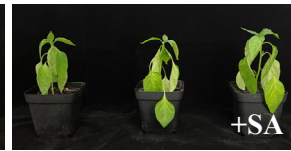

11 dpi

Supplement: Supplementary_Figures_S7_uhad213 [file supplementary_figures_s7_uhad213.pdf]

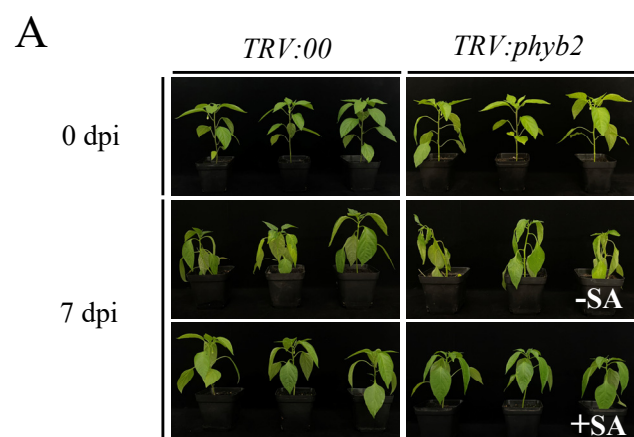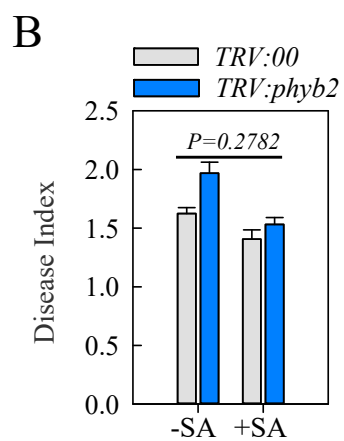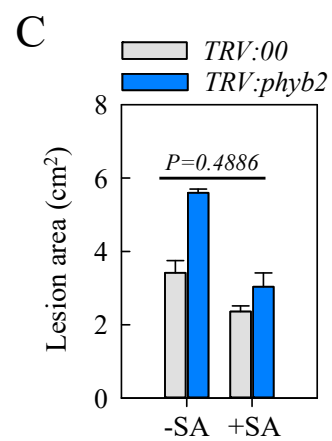

Supplement: Supplementary_Figures_S8_uhad213 [file supplementary_figures_s8_uhad213.pdf]
