# Supplementary material for: Red light induces salicylic acid accumulation by activating CaHY5 to enhance pepper resistance against Phytophthora capsici
Source: Hortic Res. 2023 Oct 17;10(11):uhad213. doi: 10.1093/hr/uhad213 (PMC10689078; doi:10.1093/hr/uhad213)
Supplement: Supplementary_Figures_S3_uhad213 [file supplementary_figures_s3_uhad213.pdf]

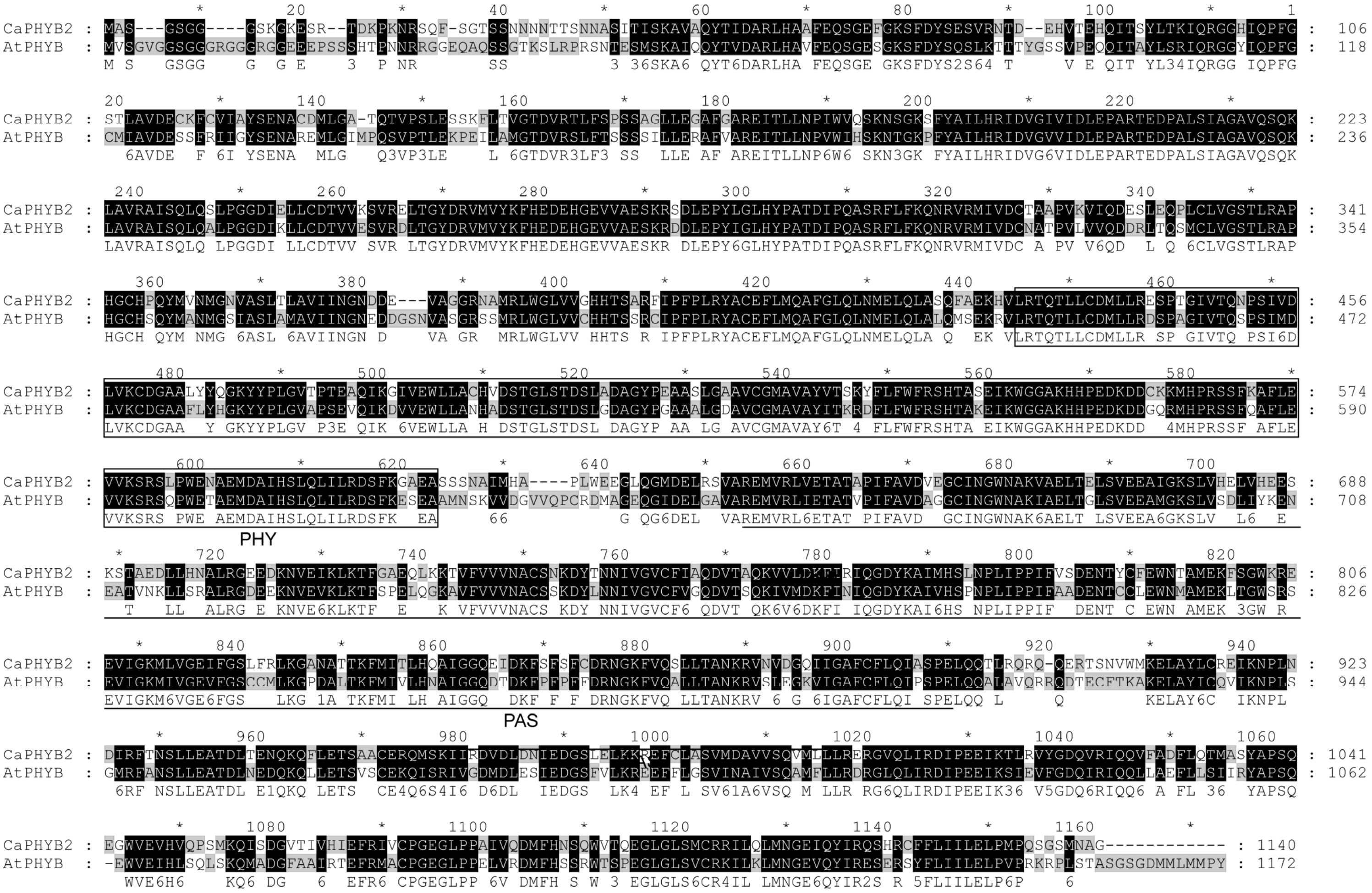

CaPHYB2
AtPHYB
PHY
PAS
