# Supplementary material for: Red light induces salicylic acid accumulation by activating CaHY5 to enhance pepper resistance against Phytophthora capsici
Source: Hortic Res. 2023 Oct 17;10(11):uhad213. doi: 10.1093/hr/uhad213 (PMC10689078; doi:10.1093/hr/uhad213)
Supplement: Supplementary_Tables_S1-2-HR-2023-625-10-9-F1_uhad213 [file supplementary_tables_s1-2-hr-2023-625-10-9-f1_uhad213.docx]

**Supplementary Tables**

**Supplementary Table S1 The primers used in this study**

| **Primer Name** | **Forward primer (5′-3′)** | **Reverse primer(5′-3′)** |
| --- | --- | --- |
| **Primers used for subcellular localization** | | |
| *CaHY5-GFP* | ccaaatcgactctagtctagaATGCAAGAGCAAGCGACAAG | tatttaaatgtcgaccccgggATTGTCCCCTTTGATATTTT |
| **Primers used for VIGS** | | |
| *TRV-CaPHYB2* | tgagtaaggttaccgaattcAATGTGGATGGCCAAATTATTGG | ggacatgcccgggcctcgagATGAACCATCCTCGATATTGTCC |
| *TRV-CaHY5* | tgagtaaggttaccgaattcAAAACAAGAGGTTAAAAAGGTTGC | ggacatgcccgggcctcgagTAGCAGGTGTGATGAGAAATTT |
| *TRV-CaPAL3* | tgagtaaggttaccgaattcACTTGGTGCCACTGTCCT | ggacatgcccgggcctcgagTAAATTCGGGCTTTCCTT |
| *TRV-CaPAL7-1* | tgagtaaggttaccgaattcGCTGTGGAAGCTGGAAAAGG | ggacatgcccgggcctcgagAGGTTGGGGTGATTTAATGTCCT |
| *TRV-CaPAL7-2* | tgagtaaggttaccgaattcCCTGCTGGATTTTGATTATGAAGC | ggacatgcccgggcctcgagCAAGTTTAGTAGTTTGGTGGTGAAA |
| **Primers used for EMSA and LUC/REN ration** | | |
| *CaHY5-p62SK* | cgctctagaactagtggatccATGCAAGAGCAAGCGACAAG | gtcgacggtatcgataagcttCTAATTGTCCCCTTTGATATTTT |
| *CaPAL3p-0800* | gtcgacggtatcgataagcttTTACTCAGGACTCTAATATA | cgctctagaactagtggatccTTTTGAAAATGATCACAAGT |
| *CaPAL7p-0800* | gtcgacggtatcgataagcttCAACTCTAGCCATTTTCTTG | cgctctagaactagtggatccCTGGAGAGGATCCATTTCAT |
| *CaHY5-MBP* | aaggatttcagaattcATGCAAGAGCAAGCGACAAG | gcaggtcgactctagaATTGTCCCCTTTGATATTTTTA |
| *CaPAL3-Cy5-EMSA* (WT) | CGTTGGATTGCACATGGGGAATCGACGATTGCGGTTGGTGA*CACGTG*GTAAACATGAAAG | CTTTCATGTTTAC*CACGTG*TCACCAACCGCAATCGTCGATTCCCCATGTGCAATCCAACG |
| *CaPAL3-Cy5-EMSA* (Mut) | CGTTGGATTGCACATGGGGAATCGACGATTGCGGTTGGTGA*CtttTG*GTAAACATGAAAG | CTTTCATGTTTACCAaaaGTCACCAACCGCAATCGTCGATTCCCCATGTGCAATCCAACG |
| *CaPAL7-Cy5-EMSA* (WT) | GGCATCAA*CAATTG*CACTAAATGGACATGTTAATGGGGATGTTGTAGCCAG | CTGGCTACAACATCCCCATTAACATGTCCATTTAGTG*CAATTG*TTGATGCC |
| *CaPAL7-Cy5-EMSA* (Mut) | GGCATCAA*CttTTG*CACTAAATGGACATGTTAATGGGGATGTTGTAGCCAG | CTGGCTACAACATCCCCATTAACATGTCCATTTAGTGCAAaaGTTGATGCC |
| **Primers used for RT-qPCR** | | |
| *qCaActin* | GACGTGACCTAACTGATAACCTGAT | CTCTCAGCACCAATGGTAATAACTT |
| *qCaHY5* | CCATTGCGGCTAGTTCTT | GCTTCTCCACCCATCTCC |
| *qCaPHYB2* | GACGCAGACTGTTCCAAGTC | AGCACCTTCAAGCAAACCTG |
| *qCaPHYB1* | GTGGTTGAGAGTGTCAGGGA | GCGTGAAGCTTGAGGAATGT |
| *qCaPR1* | CGTGAAGATGTGGGTCAATG | CCATACGGACGTTGTCCTCT |
| *qCaPR1L* | TGCTGGAGCCGTGAAGATGT | TACTGAATTACGCCACACCACTTG |
| *qCaPAL1* | ATACCCGCTCTACAGGTTCG | AGCACCATTCCACTCCTTGA |
| *qCaPAL2* | GGTTCTTGAACGCTGGAGTC | CCTTATGCCGGAGTACCCTT |
| *qCaPAL3* | ATGGTACTGCTGTTGGCTCT | GTGCGTCAAATGGTCCGTAA |
| *qCaPAL4* | AGACCTAATGCCAAGGCTGT | CGCCATACCAGAACCAACTG |
| *qCaPAL5* | AGGCCTAATTCCAAGGCTGT | CGCCATACCAGAACCAACTG |
| *qCaPAL6* | TGGGAAATGGCTGCTGAATC | TCAGAAAGCTCCACTTTAACCC |
| *qCaPAL7* | GCCCTTGCATCAATTGGGAA | GCGATTTCAGCTCCCTTGAA |
| *qCaICS1* | TCATCTTGGGACCATGCAGT | CCTTTAAGCAAGCCAGCCAT |
| *qPcActin* | TTTAGTTGGGGGTCTTGTACC | CCTCCACAACCAGCAACA |

**Supplementary Table S2 The accession numbers of PHYs used in this study**

| **Species** | **Name** | **Locus** |
| --- | --- | --- |
| **pepper** | CaPHYA | CA10g10160/Capana10g000991 |
|  | CaPHYB1 | CA01g16010/Capana01g002319 |
|  | CaPHYB2 | CA05g16200/Capana05g002211 |
|  | CaPHYE | CA02g12340/Capana02g001584 |
|  | CaPHYF | CA07g09590/Capana07g001198 |
| **Arabidopsis** | AtPHYA | [AT1G09570](https://www.arabidopsis.org/servlets/TairObject?id=27545&type=locus) |
|  | AtPHYB | AT2G18790 |
|  | AtPHYC | AT5G35840 |
|  | AtPHYD | AT4G16250 |
|  | AtPHYE | [AT4G18130](https://www.arabidopsis.org/servlets/TairObject?id=26568&type=locus) |
| **tomato** | SlPHYA | Solyc10g044670 |
|  | SlPHYB1 | Solyc01g059870 |
|  | SlPHYB2 | Solyc05g053410 |
|  | SlPHYE | Solyc02g071260.3 |
|  | SlPHYF | Solyc07g045480 |
| **Rice** | OsPHYA | Os03g51030 |
|  | OsPHYB | Os03g19590 |
|  | OsPHYC | Os03g54084 |
