# Supplementary material for: Red light induces salicylic acid accumulation by activating CaHY5 to enhance pepper resistance against Phytophthora capsici
Source: Hortic Res. 2023 Oct 17;10(11):uhad213. doi: 10.1093/hr/uhad213 (PMC10689078; doi:10.1093/hr/uhad213)
Supplement: Supplementary_Figures_S1-8-HR-2023-625-10-9-F1_uhad213 [file supplementary_figures_s1-8-hr-2023-625-10-9-f1_uhad213.docx]

**Supplementary Data**

**Supplementary Figures**


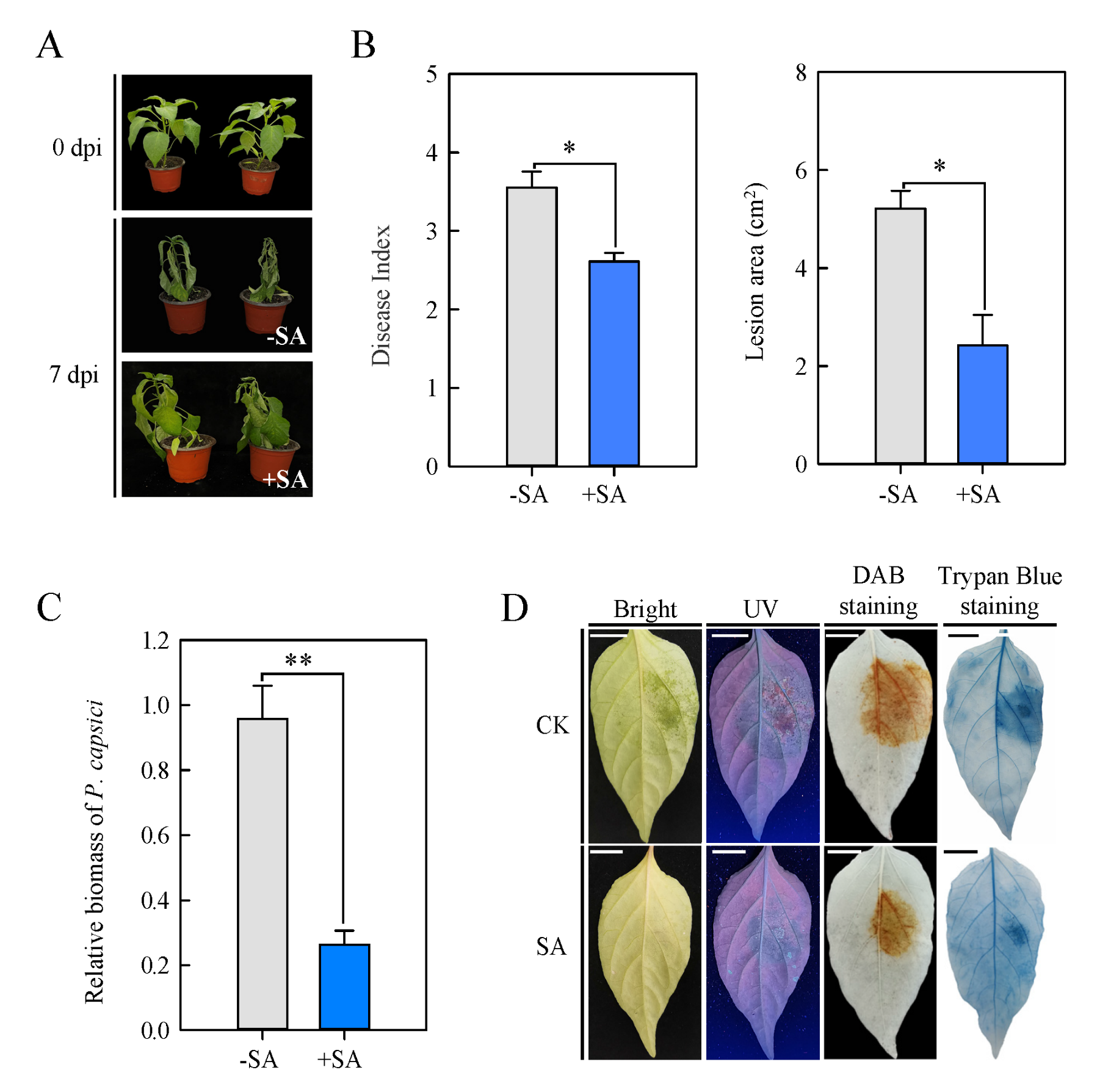


**Supplementary Figure S1. SA decreases pepper susceptibility to PCI.**

**A.** Resistance analysis of pepper plants to PCI under exogenous SA (1 mM) treatment. Photographs were acquired at 0 and 7 dpi.

**B.** SA decreases the disease index and lesion area of pepper to PCI at 7 dpi.

**C.** SA decreases the *P. capsici* biomass of pepper to PCI at 4 dpi.

**D.** The H_2_O_2_ level and cell death were analyzed in *P. capsici* infected-leaves. Images were taken at 4 dpi. Scale bar represents 1 cm.


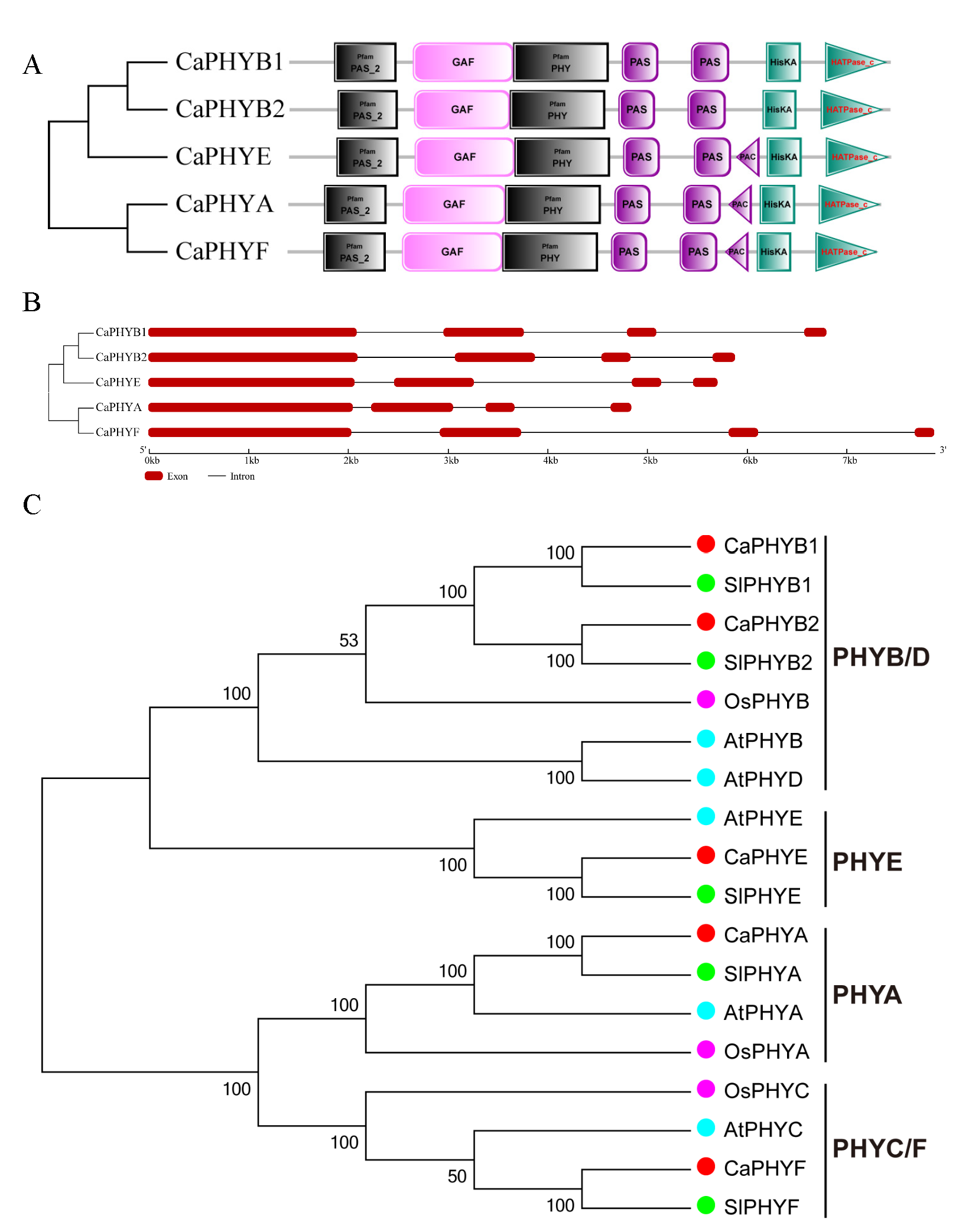


**Supplementary Figure S2. Phylogenetic analysis of putative light photoreceptor phytochromes.**

**A.** Domain distribution of PHYs in pepper proteins according to the phylogenetic relationship.

**B**. Exon-intron distribution of the pepper PHY genes. The exons and introns are presented by brownish-red boxes and black lines, respectively.

**C**. Phylogenetic analysis of the PHY proteins in different plants. The accession numbers are listed in Supplementary Table S2.


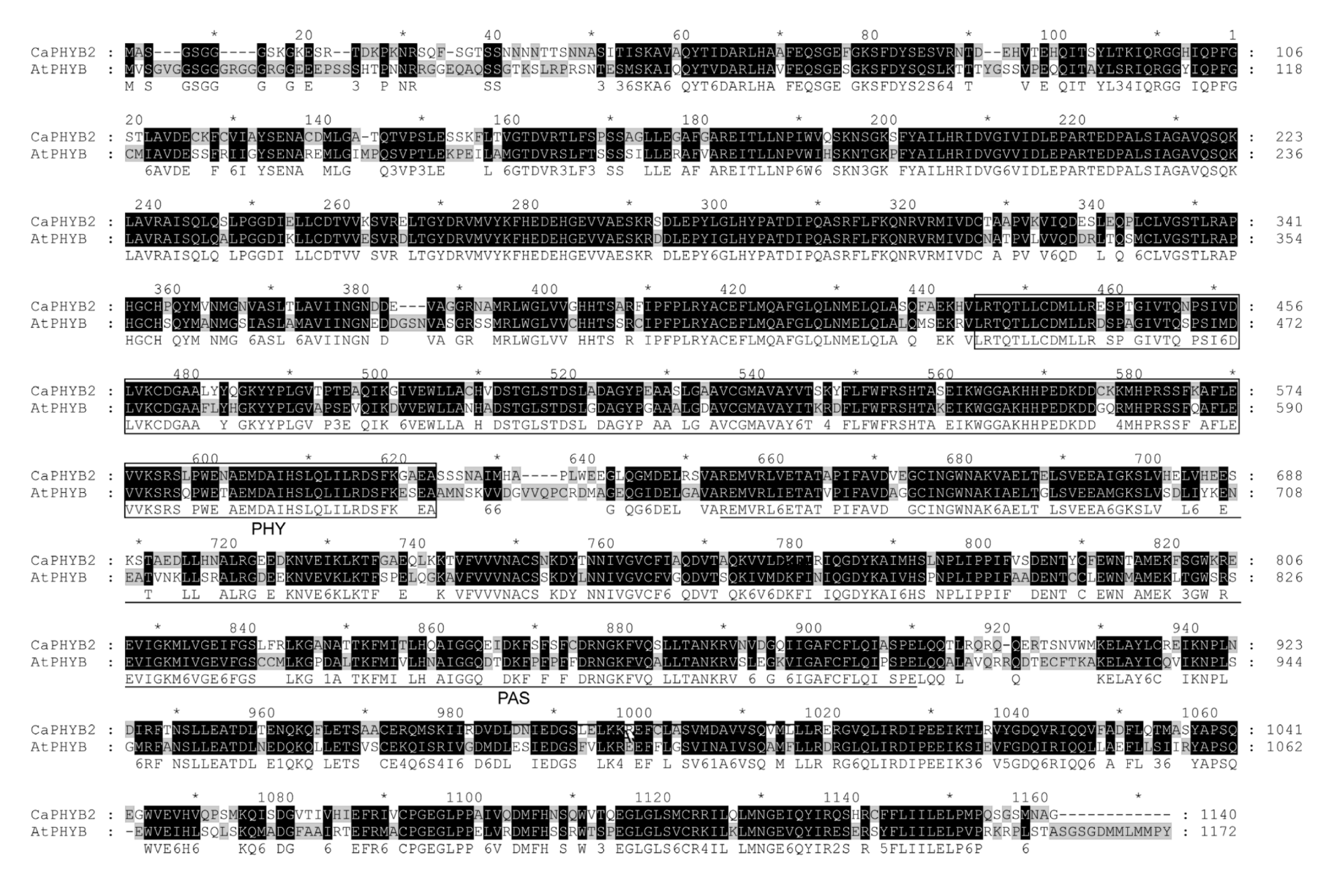


**Supplementary Figure S3. Analysis of deduced amino acid sequences of CaPHYB2 and AtPHYB.**

Multiple sequence alignment of the conserved PHY, PAS domains of PHYs from pepper CaPHYB2 (CA05g16200) and *Arabidopsis* AtPHYB ([AT2G18790](https://www.arabidopsis.org/servlets/TairObject?id=26548&type=locus)).


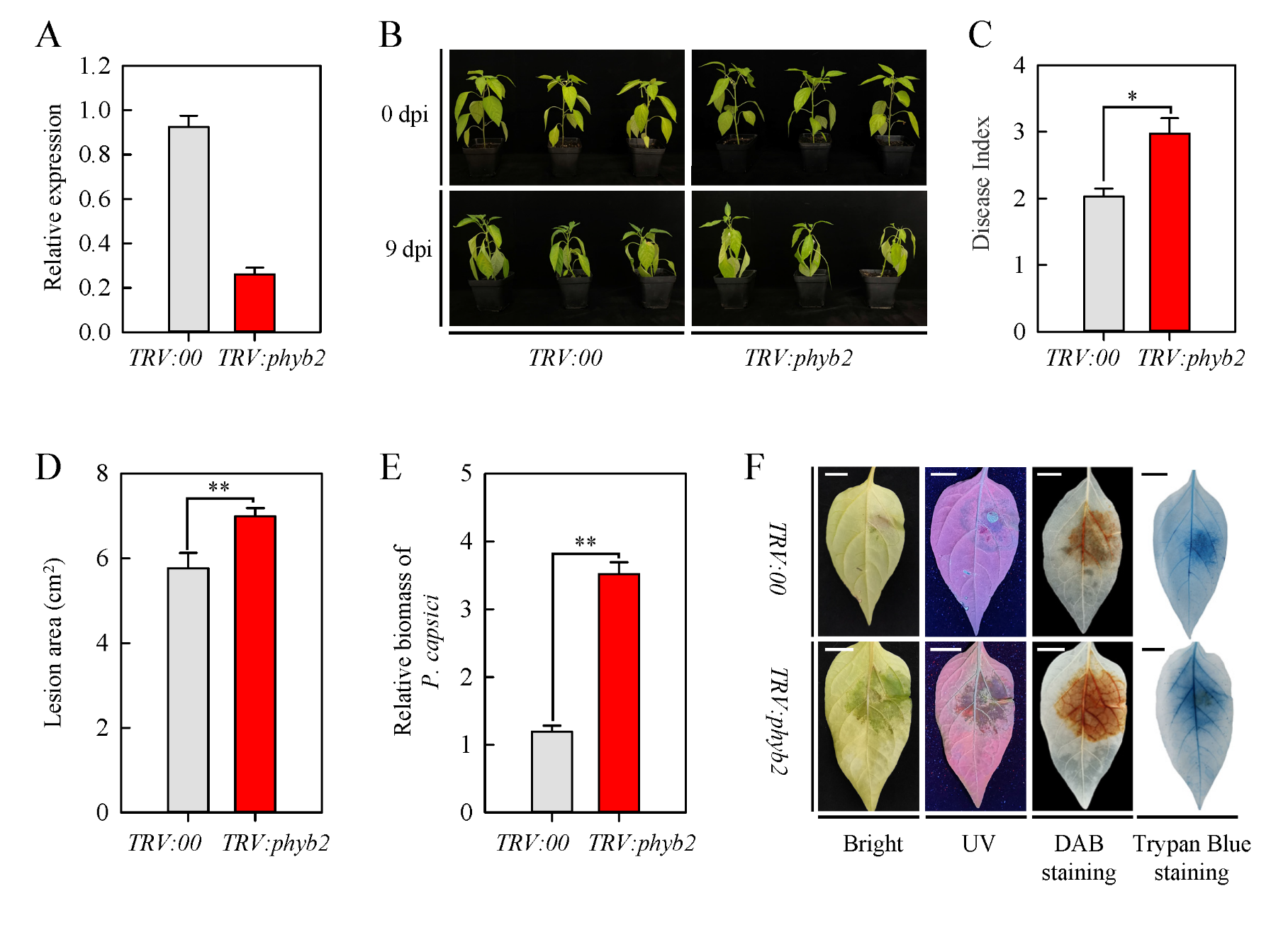


**Supplementary Figure S4. Silencing of *CaPHYB2* in pepper plants enhances susceptibility to PCI.**

**A**. Analysis of RT-qPCR data reveals that the *CaPHYB2* expression in *CaPHYB2*­silenced pepper plants is down-regulated.

**B**. Resistance level in *TRV:phyb2* and *TRV:00* (empty vector control) pepper plants at 9 dpi with *P. capsici*.

**C**-**E**. Disease index (**C**, 9 dpi), lesion area (**D**, 4 dpi), and *P. capsici* biomass (**E**, 4 dpi) in *TRV:phyb2* and *TRV:00* during PCI in pepper*.* Data represent the mean ± SE (n=3).

**F**. Increase in the H_2_O_2_ level and cell death in *TRV:phyb2* pepper leaves compared with those in control (*TRV:00*) leaves at 4 dpi with *P. capsici*. Scale bar represents 1 cm.


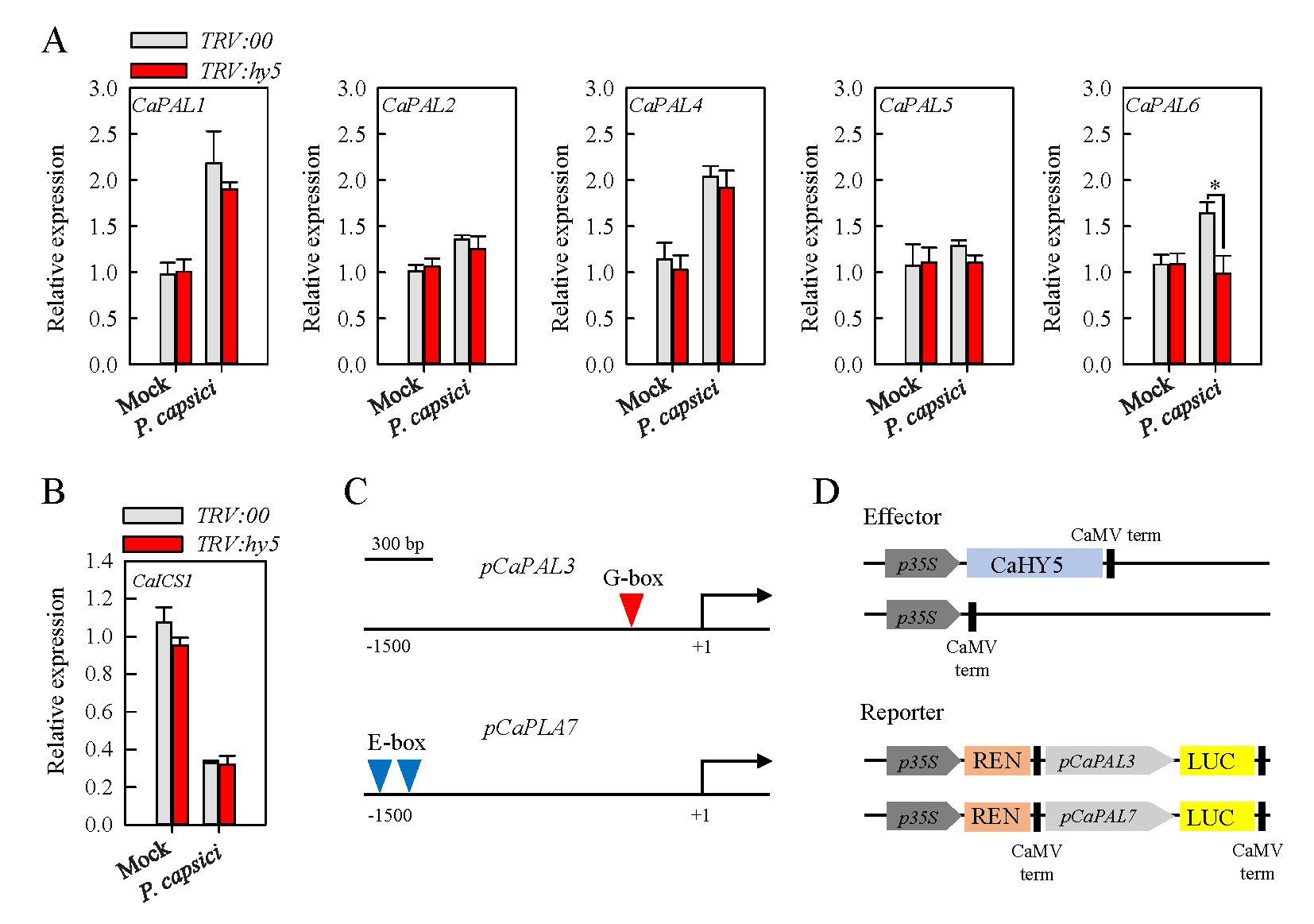


**Supplementary Figure S5. Expression analysis of SA biosynthesis-related *CaPALs* and *CaICS1* genes in *CaHY5-*silenced pepper leaves.**

**A.** Expression levels of *CaPAL1*, *CaPAL2*, *CaPAL4*, *CaPAL5*, and *CaPAL6* analyzed by RT-qPCR in *TRV:hy5* and *TRV:00* pepper leaves at 4 dpi with *P. capsici*.

**B.** Expression levels of *CaICS1* analyzed by RT-qPCR in *TRV:hy5* and *TRV:00* pepper leaves at 4 dpi with *P.capsici*. Data represent the mean ± SE (n=3).

**C.** Analysis of G-Box and E-box elements in *CaPAL3* and *CaPAL7* promoters. The triangles indicate the sequence position for the G-Box or E-box.

**D**. Structural schematic diagrams of vector constructs used for the dual-luciferase reporter assay.


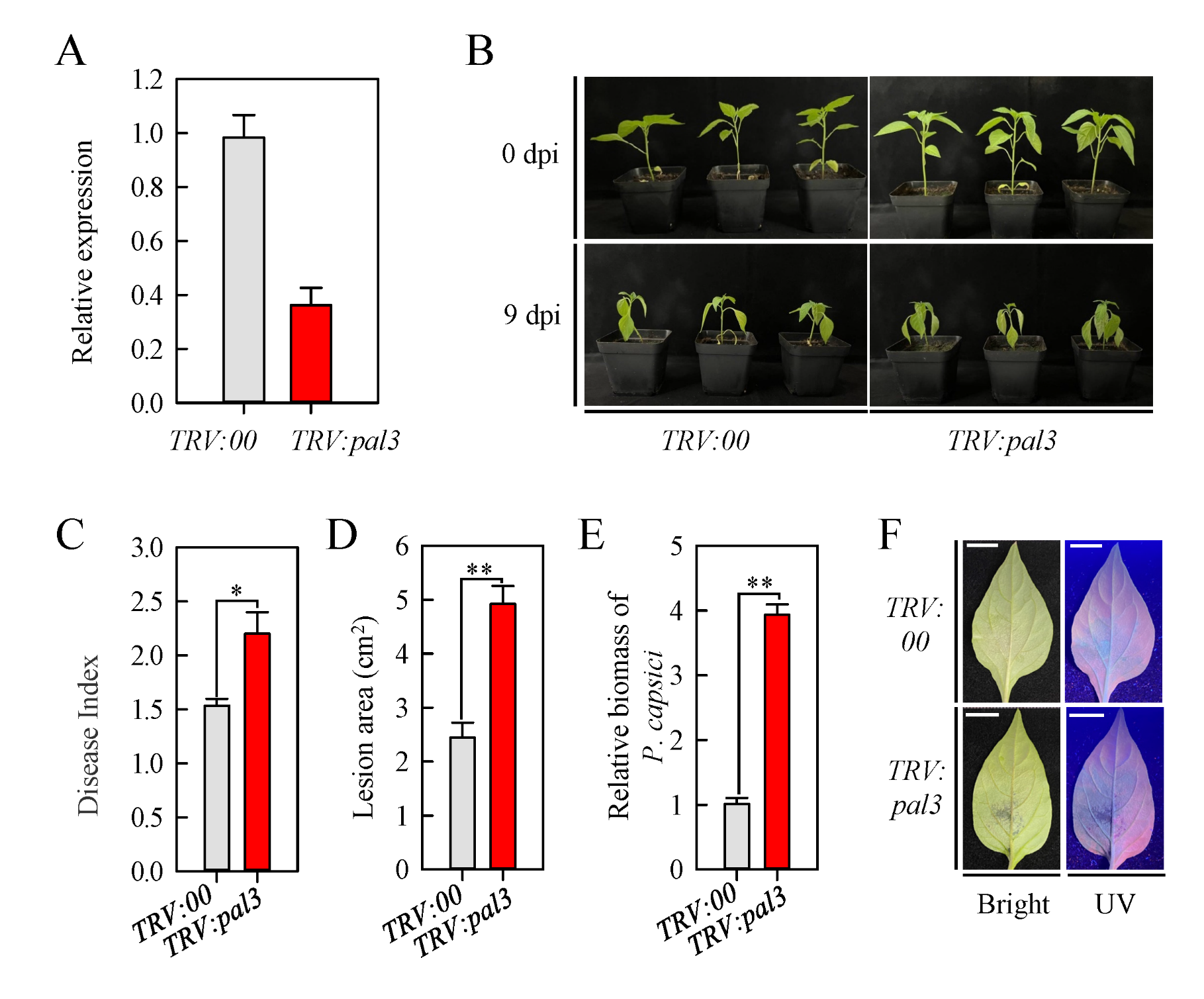


**Supplementary Figure S6. Silencing of *CaPAL3* in pepper plants enhances susceptibility to PCI.**

**A**. Analysis of RT-qPCR data reveals that the *CaPAL3* expression in *CaPAL3*­silenced pepper plants is down-regulated.

**B**. Resistance level in *TRV:pal3* and *TRV:00* (empty vector control) pepper plants at 9 dpi with *P. capsici*.

**C**-**E**. Disease index (**C**, 9 dpi), lesion area (**D**, 4 dpi), and *P. capsici* biomass (**E**, 4 dpi) in *TRV:pal3* and *TRV:00* during PCI in pepper*.* Data represent the mean ± SE (n=3).

**F**. Resistance level in *TRV:pal3* and *TRV:00* pepper plants at 4 dpi with *P. capsici*. Scale bar represents 1 cm.


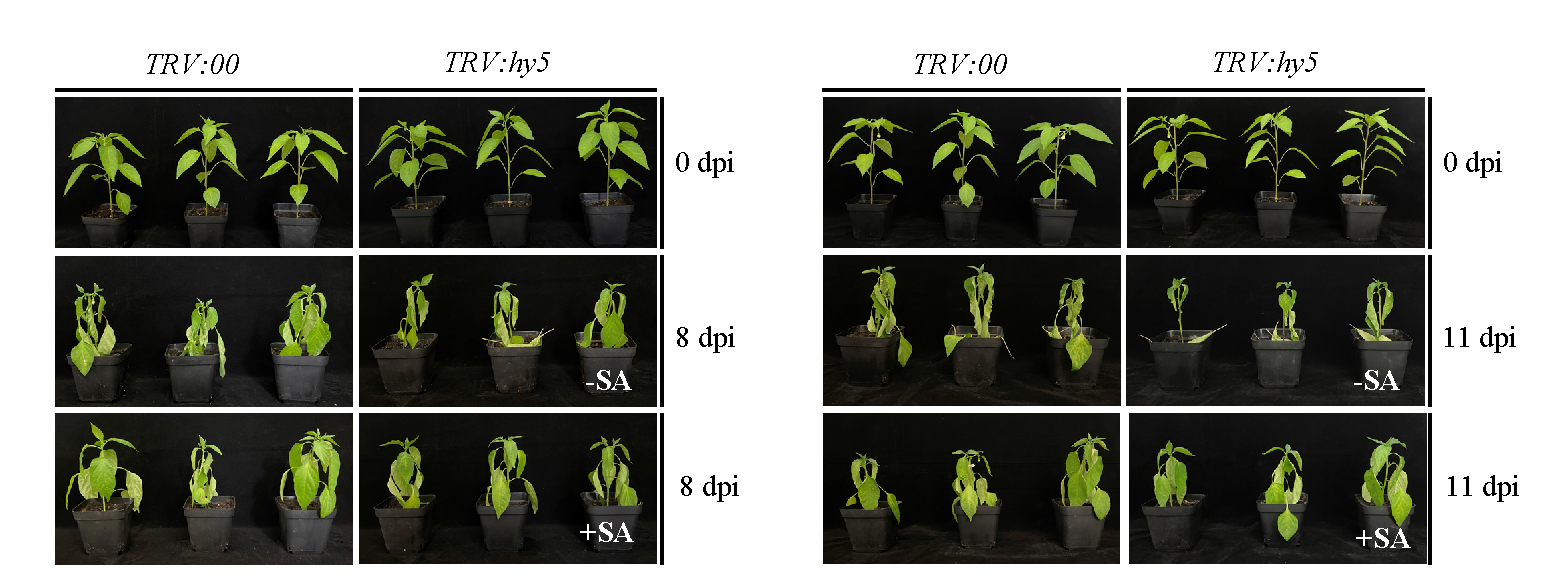


**Supplementary Figure S7. Resistance levels of *TRV:hy5* pepper plants to PCI were enhanced by exogenous SA application.** Photographs were acquired at 0, 8, and 11 dpi.


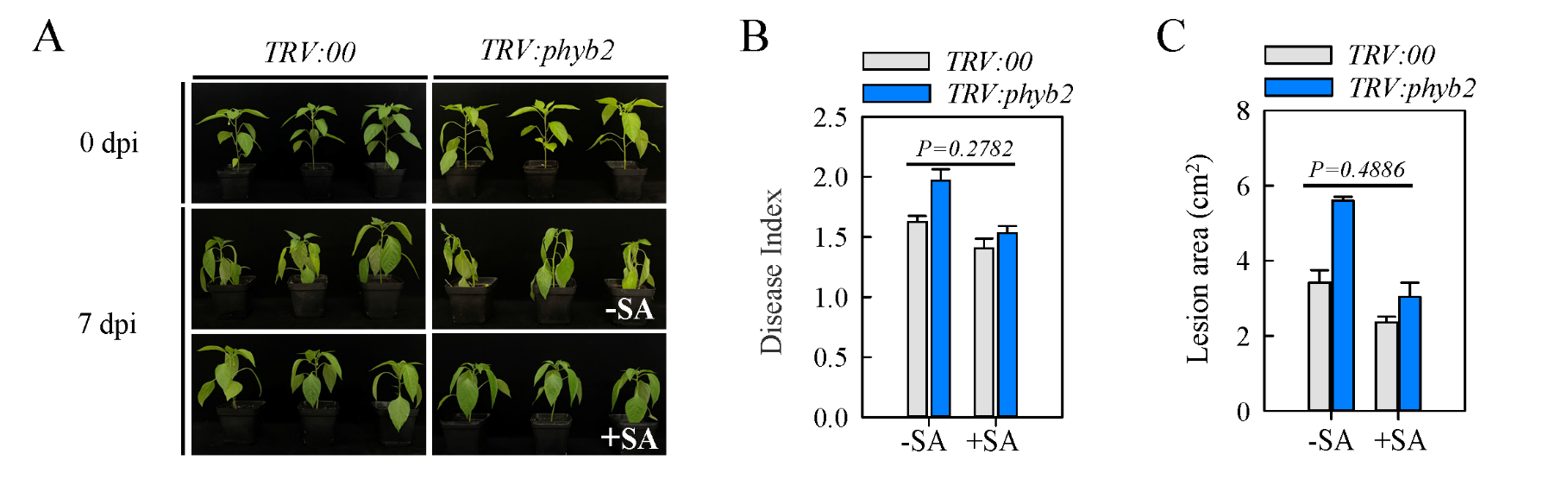


**Supplementary Figure S8. Application of exogenous SA restores resistance levels of *TRV:phyb2* pepper plants to PCI.**

**A**. Resistance analysis of *TRV:phyb2* and *TRV:00* pepper plants to PCI upon exogenous application of SA (1 mM). Photographs were acquired at 7 dpi.

**B and C**. Disease index (7 dpi ) and lesion area (4 dpi) in *TRV:phyb2* and *TRV:00* pepper plants to PCI were decreased by exogenous SA (1 mM) treatment.
